# Full observability and estimation of unknown inputs, states and parameters of nonlinear biological models

**DOI:** 10.1098/rsif.2019.0043

**Published:** 2019-07-03

**Authors:** Alejandro F. Villaverde, Nikolaos Tsiantis, Julio R. Banga

**Affiliations:** 1 Bioprocess Engineering Group, IIM-CSIC, Vigo, Galicia 36208, Spain; 2 Department of Chemical Engineering, University of Vigo, Vigo, Galicia 36310, Spain

**Keywords:** identifiability, system identification, parameter estimation, observability, input reconstruction, dynamic modelling

## Abstract

In this paper, we address the system identification problem in the context of biological modelling. We present and demonstrate a methodology for (i) assessing the possibility of inferring the unknown quantities in a dynamic model and (ii) effectively estimating them from output data. We introduce the term Full Input-State-Parameter Observability (FISPO) analysis to refer to the simultaneous assessment of state, input and parameter observability (note that parameter observability is also known as identifiability). This type of analysis has often remained elusive in the presence of unmeasured inputs. The method proposed in this paper can be applied to a general class of nonlinear ordinary differential equations models. We apply this approach to three models from the recent literature. First, we determine whether it is theoretically possible to infer the states, parameters and inputs, taking only the model equations into account. When this analysis detects deficiencies, we reformulate the model to make it fully observable. Then we move to numerical scenarios and apply an optimization-based technique to estimate the states, parameters and inputs. The results demonstrate the feasibility of an integrated strategy for (i) analysing the theoretical possibility of determining the states, parameters and inputs to a system and (ii) solving the practical problem of actually estimating their values.

## 1. Introduction

Many biological processes can be adequately described by dynamic models consisting of a set of ordinary differential equations (ODEs) [1]. The components of the model equations can be classified according to their dependence on time (constant or time-varying) and to the knowledge that the modeller or user possesses about them (known or unknown). The system identification problem is to determine the unknown quantities present in the model equations from measured data. Several subproblems and methodologies can be considered as part of the general reverse engineering problem [2–4].

*Identifiability* analysis assesses the possibility of determining the parameter values from output measurements [1,4,5]. A *parameter* is defined as an unknown constant appearing in the model equations. Likewise, *observability* describes the ability to infer the model states from the model output [1,6,7]. A *state* is a dynamic variable whose time dependence is described by one of the differential equations of the model. Finally, a model may have *unknown inputs*, which can also be seen as external *disturbances* or *time-varying parameters*. The possibility of inferring them is sometimes called *input observability* [8,9], *input reconstructibility* [10] or *system invertibility* [11–14].

These properties can be analysed from *structural* and *practical* viewpoints. Structural identifiability refers to the theoretical possibility of determining parameter values. It is completely defined by the model equations and the input–output mapping, and it does not take into account limitations arising from data quantity or quality; symmetries in model equations are common sources of structural unidentifiability [6,7,15,16]. By contrast, practical or numerical identifiability (sometimes also called estimability) refers to the quantification of the uncertainty in parameter estimates taking into account the actual data used for calibration [1,4]. Structural identifiability is a necessary but not sufficient condition for practical or numerical identifiability. The previous sentences have discussed the structural and practical viewpoints for the parameter identifiability property; a similar distinction between structural and practical can be made for state observability and input reconstructibility.

The analysis of structural properties must be performed *a priori*, i.e. before attempting to calibrate or to use a model in any way, in order to detect any structural issues and distinguish them from numerical problems, which should be dealt with differently. This analysis is important not only because the biological interpretations of structurally unidentifiable parameters are not valid, but also because predictions about unmeasured states and inputs of the system may also be wrong if the model has structural deficiencies [7,17–19]. Such errors can have serious consequences; for example, in the context of biomedical applications, they may lead to wrong diagnoses or sub-optimal treatments [20–22].

It should be noted that the three aforementioned properties are sometimes simply called observability (i.e. state observability, parameter observability, input observability). In fact, a model parameter can be considered as a state variable whose time derivative is zero, so identifiability can be simply considered as a subcase of observability [6,7,23]. This use of the term observability is common in the systems and control literature. However, in biological sciences the use of the term identifiability is more common than parameter observability. Historically, the biological modelling community has paid considerable attention to the problem of parametric identifiability, and many techniques for identifiability analysis have been developed in this community, despite being applicable in other contexts [1,4,5,15,24–26]. This is due to the challenging nature of the parameter identification problem in biology compared to many engineering applications, which has motivated the development of new methods in this area. Thus, although we can use the term observability to refer to all the aforementioned properties, we also mention identifiability and reconstructibility to avoid confusion. To make the distinction explicit, in this paper we introduce the term Full Input-State-Parameter Observability (FISPO). We use it for characterizing the property of a model for which it is theoretically possible to determine the values of its unknown parameters, inputs and states, without requiring assumptions about the knowledge of some variables in order to determine the others. This is emphasized here because, if a model has unknown parameters *and* unmeasured states *and* unknown inputs, the three aforementioned properties (identifiability, observability and reconstructibility) are interrelated, and it is not possible to study one independently of the others.

The question of unknown input observability, also called reconstructibility or invertibility, was initially studied in the literature for linear systems [8–11]. A number of works have addressed the problem of analysing parameter structural identifiability and input observability jointly. The differential algebra approach [27] has been applied to the analysis of certain nonlinear models containing the so-called ‘time-varying parameters’ [28,29]. The differential algebra algorithms currently available for this analysis can be applied to polynomial or rational models of relatively small size. As an alternative, Martinelli has recently proposed to address this problem from a differential geometry viewpoint [30–32], presenting an extended observability rank condition [33] that can be applied to systems with ‘unknown inputs’ (note the different terminology). For this condition to be applicable, the model dynamics can be nonlinear in the states but must be linear with respect to the inputs, both known and unknown. A related method was presented in [34]. The algorithms in [33,34] analyse observability of states and parameters in the presence of an unknown input, but not the observability of the input itself (although they could conceivably be modified for this purpose).

Once the FISPO of a model has been analysed, and assuming that the model is fully observable, a question naturally arises: how to effectively estimate its unknown parameters and inputs? (Once the inputs and parameters are determined, it is straightforward to obtain the model states by simulation, as long as any unknown initial conditions are included in the unknown parameter vector for estimation purposes.) In other words, how to deal with the *estimation* problem, once the *observability* problem (i.e. the FISPO analysis) has been solved? In a previous work [35], we addressed the estimation problem with an optimal tracking approach, with which we inferred both the time-dependent inputs and the time-invariant parameters simultaneously from noisy dynamic data. We provided an implementation of this approach along with examples, as an add-on for the AMIGO2 toolbox [36]. An alternative open source software for input reconstruction is Data2Dynamics/d2d [37]. We remark that in [35] we did not consider the reconstructibility problem, i.e. we did not assess the FISPO of the models used as case studies, since we did not have the tools for such analysis. Likewise, other related works have considered different instances of input reconstruction problems addressing the practical estimation problem [38–42]. In particular, Schelker *et al.* [39] considered uncertainty in the input measurements within the general parameter estimation (PE) formulation, while Kaschek *et al.* [38] used a calculus of variations-based approach and Trägårdh *et al.* [40] formulated the input reconstruction as a Bayesian inference problem. Furthermore, Engelhart *et al.* [41,42] formulated a more general reconstruction problem in order to estimate not only unknown inputs but also missed and erroneous interactions. However, all the aforementioned papers assumed or overlooked the theoretical reconstructibility of the full system without providing any methodology to analyse it. As a notable exception, Trägårdh *et al.* [43] analysed the input observability of a pharmacokinetic model using the Taylor series expansion [44] before estimating the input. Unlike in the FISPO analysis proposed in the present work, Trägårdh *et al.* analysed input observability and parameter identifiability independently, without taking into account possible interaction effects.

**Figure 1.**
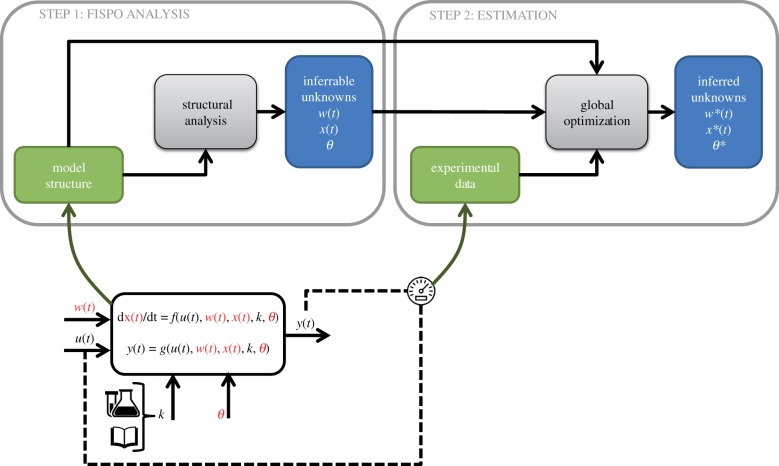
Workflow of the methodology followed in this paper. There are two main steps: (1) an initial structural analysis (FISPO) determines which unknown inputs (*w*(*t*)), parameters (*θ*) and states (*x*(*t*)) can be inferred and (2) a numerical estimation procedure recovers the values of the inferrable variables via optimization. The information required by the methodology is included in the green boxes, the grey boxes denote computational procedures and the blue boxes are the intermediate and final results of the methodology. The lower part of the figure shows the model structure components: dynamic states *x*(*t*), measured outputs *y*(*t*) (typically a subset of the states, but may also be a function of them), known constants *k* (either taken from the literature or previously measured), unknown constant parameters *θ*, known inputs *u*(*t*) and unknown inputs *w*(*t*). The model unknowns (unknown inputs, parameters and states) are coloured in red. Note that a state may be actually known if it is being measured, but this knowledge is included in the model as an output, *y*_*i*_(*t*) = *x*_*j*_(*t*), hence states are coloured in red. (Online version in colour.)

In the present work, we address both the (theoretical) observability and the (practical) estimation problem. We first determine if it is possible to infer the unmeasured states, inputs, and parameters of nonlinear models (that is, the properties individually known as observability, reconstructibility and identifiability) from a structural point of view. To perform this analysis, which we have called FISPO, we adopt a differential geometry approach. To this end, we have extended a recent computational tool [45] in a way that is in principle applicable to any nonlinear ODE model with unknown inputs, as long as its dynamic and output equations are analytic. We demonstrate its use by applying it to three case studies from physiology, viral dynamics and synthetic biology. Analysing the FISPO *a priori* allows detection of structural issues and distinguishing them from other possible causes of failure in the estimation, such as limitations of the optimization algorithm or insufficient information in the calibration data. Such types of deficiencies can cause *numerical* or *practical* issues, which are fundamentally different to *structural* ones and must be dealt with in a different way. Thus, the FISPO analysis yields a theoretical result that is necessary but not always sufficient in practice. Hence after analysing it for each case study we assess the possibility of actually estimating the unmeasured variables from output measurements. To this end, we define numerical scenarios generating pseudo-experimental data, covering different types of unmeasured inputs: piecewise constant, ramp and sinusoidal. Next, we apply a hybrid optimization method to recover the values of the model unknowns. For all case studies, the methodology manages to estimate the unknown inputs without prior knowledge about their shape, along with the parameters and states. The workflow of the methodology, which addresses both the theoretical and the practical identification problems, is shown in figure 1. We finish the paper by discussing the strengths and limitations of our approach and outlining future work.

## 2. Methods

### 2.1. Notation and definitions

In this paper, we consider nonlinear ODE models of the following general form:

2.1 $$\text{model }M_{1}:\;\left\{ \begin{array}{c} \dot{x}(t)=f(u(t),w(t),x(t),k,\theta ),\\ y(t)=g(u(t),w(t),x(t),k,\theta ) \end{array}\right.$$

where $x(t)\in R^{n_{x}}$ is the state variables vector, $u(t)\in R^{n_{u}}$ the known inputs vector, $w(t)\in R^{n_{w}}$ the unknown inputs vector, $\theta \in R^{n_{\theta }}$ the unknown parameter vector, $k\in R^{n_{k}}$ the known constants vector, $y(t)\in R^{n_{y}}$ the output vector, and *f* and *g* are analytic vector functions. The input vectors *u*(*t*) and *w*(*t*) are in principle assumed to be smooth, i.e. infinitely differentiable functions. Figure 1 shows the components of the mathematical models considered in this paper. Note that, in order to make the distinction between known and unknown parameters explicit, we have included a vector of known constants, *k*, in the equations. In the remainder of this paper, we will drop this dependency on *k* from the notation.

A parameter *θ*_*i*_ of a nonlinear model *M*_1_ (2.1) is *structurally locally identifiable* (s.l.i.) if for almost any parameter vector $\theta^{*}\in R^{n_{\theta }}$ there is a neighbourhood $N(\theta^{*})$ in which the following holds:

2.2 $$\hat{\theta }\in N(\theta^{*})\;\text{and}\;y(t,\hat{\theta })=y(t,\theta^{*})\Rightarrow \hat{\theta_{i}}=\theta_{i}^{*}.$$

If this relationship does not hold in any neighbourhood of θ*, *θ*_*i*_ is *structurally unidentifiable* (s.u.). If all the parameters in a model are s.l.i., the model is also said to be s.l.i. However, if a model has one or more s.u. parameters, it is said to be s.u.

Similarly, a state *x*_*i*_(*τ*) is said to be *observable* if it can be determined from the output *y*(*t*) and any known inputs *u*(*t*) of the model in the interval *t*_0_ ≤ *τ* ≤ *t* ≤ *t*_*f*_, for a finite *t*_*f*_ [1]. Otherwise, it is *unobservable*. A model is observable if all its states are observable. We use a similar definition for unknown inputs: we say that *w*_*i*_(*τ*) is *reconstructible* if it can be determined from *y*(*t*) and *u*(*t*) in *t*_0_ ≤ *τ* ≤ *t* ≤ *t*_*f*_, for a finite *t*_*f*_.

To refer to the joint property of structural identifiability of all parameters, observability of all states and reconstructibility of all unknown inputs, we use the term *Full Input-State-Parameter Observability*, and abbreviate it as FISPO. We use this acronym both as a noun (to name the property) and as an adjective (to refer to the model that fulfils this property); in the latter case, it stands for Full Input-State-Parameter Observable. The FISPO property is formally defined as follows:

**Definition 2.1.**

FISPO. Consider a model *M*_1_ given by (2.1). Let *z*(*t*) = [*x*(*t*), *θ*, *w*(*t*)] be the vector of unknown model quantities (i.e. states, parameters and inputs), with $z(t)\in R^{n_{x}+n_{\theta }+n_{w}}$, and let us denote each element of *z*(*t*) at time *τ* as *z*_*i*_(*τ*). We say that *M*_1_ has the *FISPO* property if every *z*_*i*_(*τ*) can be determined from the output *y*(*t*) and any known inputs *u*(*t*) of the model in the interval *t*_0_ ≤ *τ* ≤ *t* ≤ *t*_*f*_, for a finite *t*_*f*_. Thus, *M*_1_ is FISPO if, for every *z*_*i*_(*τ*), for almost any vector z*(*τ*) there is a neighbourhood $N(z^{*}(\tau ))$ in which the following holds:

2.3 $$\begin{array}{rl} & \hat{z}(\tau )\in N(z^{*}(\tau ))\;\text{and}\\ & \;y(t,\hat{z}(\tau ))=y(t,z^{*}(\tau ))\Rightarrow {\hat{z}}_{i}(\tau )=z_{i}^{*}(\tau ). \end{array}$$

### 2.2. Structural identifiability and observability: the differential geometry framework

State observability and parametric structural identifiability can be jointly studied from a differential geometry viewpoint. In this approach, the parameters are considered as constant state variables. Observability of said state variable is equivalent to structural local identifiability of the corresponding parameter. By recasting, the parameters as state variables whose dynamics are zero [23,46,47] we obtain an augmented state $\tilde{x}$ of dimension $n_{\tilde{x}}=n_{x}+n_{\theta }$:

2.4 $$\tilde{x}(t)=\left[\begin{array}{c} x(t)\\ \theta \end{array}\right]\;\text{and}\;\dot{\tilde{x}}(t)=\left[\begin{array}{c} f(\tilde{x}(t),u(t),w(t))\\ 0 \end{array}\right].$$

We can now use extended Lie derivatives to build an observability–identifiability matrix. To illustrate the approach, let us consider first the case without unknown inputs, i.e.

2.5 $$\text{model }M_{2}:\;\left\{ \begin{array}{c} \dot{x}(t)=f(\tilde{x}(t),u(t)),\\ y(t)=g(\tilde{x}(t),u(t)). \end{array}\right.$$

The *extended Lie derivative* of a function $g(\tilde{x},u)$ with respect to $f(\tilde{x},u)$ is defined by [48]

2.6 $$L_{f}g(\tilde{x},u)=\frac{\partial g(\tilde{x},u)}{\partial \tilde{x}}f(\tilde{x},u)+\sum_{\;j=0}^{\;j=\infty }\frac{\partial g(\tilde{x},u)}{\partial u^{\left(j\right)}}u^{\left(j+1\right)},$$

where *u*^(*j*)^ is the *j*th time derivative of the input *u*. The Lie derivatives of order higher than one are obtained as

2.7 $$L_{f}^{i}g(\tilde{x},u)=\frac{\partial L_{f}^{i-1}g(\tilde{x},u)}{\partial \tilde{x}}f(\tilde{x},u)+\sum_{\;j=0}^{\;j=\infty }\frac{\partial L_{f}^{i-1}g(\tilde{x},u)}{\partial u^{\left(j\right)}}u^{\left(j+1\right)}.$$

Since the output may depend on the input, but not on its derivatives, the partial derivatives of the output with respect to input derivatives are zero, and the infinite summation in equation (2.6) can actually be truncated in *j* = 0. Likewise, the *i*th Lie derivative, *L*^*i*^_*f*_*g*, may contain input derivatives only up to order *i*, if the output depends directly on the input, and up to a lower order otherwise. Therefore, it is not necessary to calculate the infinite summation in equation (2.7), but instead

2.8 $$L_{f}^{i}g(\tilde{x},u)=\frac{\partial L_{f}^{i-1}g(\tilde{x},u)}{\partial \tilde{x}}f(\tilde{x},u)+\sum_{\;j=0}^{\;j=i}\frac{\partial L_{f}^{i-1}g(\tilde{x},u)}{\partial u^{\left(j\right)}}u^{\left(j+1\right)}.$$

The observability–identifiability matrix, $O_{I}(\tilde{x},u)$, is

2.9 $$O_{I}(\tilde{x},u)=\left(\begin{array}{c} \frac{\partial }{\partial \tilde{x}}g(\tilde{x},u)\\ \frac{\partial }{\partial \tilde{x}}(L_{f}g(\tilde{x},u))\\ \frac{\partial }{\partial \tilde{x}}(L_{f}^{2}g(\tilde{x},u))\\ \vdots \\ \frac{\partial }{\partial \tilde{x}}(L_{f}^{n_{\tilde{x}}-1}g(\tilde{x},u)) \end{array}\right).$$

**Theorem 2.2.**

*Nonlinear Observability–Identifiability Condition* (*OIC*). *If a model* *M*_2_ *defined by* (*2.5*) *satisfies* $\text{rank}(O_{I}({\tilde{x}}_{0},u))=n_{x}+n_{\theta }$, *with* $O_{I}({\tilde{x}}_{0},u)$ *given by* (*2.9*) *and* ${\tilde{x}}_{0}$ *being a* (*possibly generic*) *point in the augmented state space*, *then the model is locally observable and locally structurally identifiable in a neighbourhood* $N({\tilde{x}}_{0})$ *of* ${\tilde{x}}_{0}$ *[*48*].*

It should be noted that $O_{I}$ may have full rank even when built with less than (*n*_*x*_ + *n*_*θ*_ − 1) Lie derivatives. In practice, this means that it is often more efficient to build $O_{I}$ recursively and calculate $\text{rank}(O_{I})$ after adding each Lie derivative, which allows for an early termination of the procedure if full rank is achieved (in which case the OIC is fulfilled) or if the rank stops increasing (the OIC is not fulfilled).

If the OIC does not hold, there is at least one unobservable state (or an unidentifiable parameter). They can be found by removing each of the columns of $O_{I}$ and recalculating its rank. If the rank does not change after removing the *i*th column, the *i*th variable (which may be a parameter or a state) is structurally unidentifiable (or unobservable) [49].

Some models may require the inputs to be sufficiently exciting in order to be identifiable. The necessary input can be characterized to a certain extent by setting to zero in *O*_*I*_ the derivatives of the input *u* of order higher than a given one and recalculating the rank [50]. This enables, for example, detection of whether a model is unidentifiable with a constant input but becomes identifiable with a ramp. In some cases, several experiments performed under different conditions can yield better observability properties than a single experiment. This scenario can be analysed with the multi-experiment setting described in [50]. In it, the model is modified to include as many replicates of the state, output and input vectors as experiments, increasing the dimension of the $\left(O_{I}\right)$ matrix. It may also be convenient to perform a single experiment with several intervals, in each of which the input is infinitely differentiable. Such *piecewise* infinitely differentiable inputs may also be approximated by the multi-experiment setting for the purpose of observability analysis. To this end, each of the time intervals in which the input is infinitely differentiable is considered as a different experiment. Note however that, since the instantaneous transitions between differentiable intervals cannot be included in such analysis, this approximation might lead to errors of unknown magnitude.

The differential geometry approach yields results that are valid almost everywhere, i.e. for all values of the system variables except for a set of measure zero. Therefore, this type of analysis does not consider inputs such as delta functions, which are zero everywhere except in discrete time points. In any case, it should be noted that delta functions are not frequently used in biological systems modelling.

### 2.3. Extending the generalized observability analysis to account for unknown inputs

Let us now consider the case in which there are unmeasured inputs to the system, *w*(*t*), the value of which is unknown. These inputs can also be seen as external disturbances or, alternatively, as unknown, time-varying parameters. That is, we wish to study models such as *M*_1_ (2.1), which requires extending the formulation presented in §2.2, since it is only applicable to models such as *M*_2_ (2.5). To this end, we augment the state vector so as to include also the unknown inputs

2.10 $$\tilde{x}(t)=\left[\begin{array}{c} x(t)\\ \theta \\ w(t) \end{array}\right]\;\text{and}\;\dot{\tilde{x}}(t)=\left[\begin{array}{c} f(\tilde{x}(t),u(t))\\ 0\\ \dot{w}(t) \end{array}\right].$$

This transforms a model of the form (2.1) in another of the form (2.5), making it seemingly amenable to the technique described in §2.2. However, there is an obvious caveat: the expression of $\dot{w}(t)$ is of course unknown and, as Lie derivatives are calculated to build $O_{I}$, the order of derivatives of *w*(*t*) that may appear in $O_{I}$ increases; the *i*th Lie derivative, $L_{f}^{i}g(\tilde{x},u)$, may contain derivatives up to *w*^(*i*)^. To account for this, we also include those derivatives in the augmented state vector, that is

2.11 $$\tilde{x}(t)=\left[\begin{array}{c} x(t)\\ \theta \\ w(t)\\ \dot{w}(t)\\ \ddot{w}(t)\\ \vdots \\ w^{\left(i-1\right)}(t)\\ w^{\left(i\right)}(t) \end{array}\right]\;\text{and}\;\dot{\tilde{x}}(t)=\left[\begin{array}{c} f(\tilde{x}(t),u(t))\\ 0\\ \dot{w}(t)\\ \ddot{w}(t)\\ w^{\ldots }(t)\\ \vdots \\ w^{\left(i\right)}(t)\\ w^{\left(i+1\right)}(t) \end{array}\right],$$

with $\tilde{x}\in R^{n_{\tilde{x}}}$, $n_{\tilde{x}}=n_{x}+n_{\theta }+n_{w}\cdot (i+1)$.

**Theorem 2.3.**

*Full Input, State, and Parameter Observability Condition*: *a model* *M*_1_ *given by* (*2.1*) *is FISPO according to definition* *2.1* *if*, *adopting the state augmentation of equation* (*2.11*), *the resulting generalized observability matrix* (*2.9*) *is such that* $\text{rank}(O_{I}(\tilde{x},u))=n_{\tilde{x}}$.

**Proof.**

A model *M*_1_ of the form (2.1) can be recast into a model of the form (2.5) using the state augmentation (2.11). The augmented state vector of this model includes the original state variables, the parameters and the unknown inputs as well as their first *i* time derivatives. Thus, it is possible to build the generalized observability matrix as in (2.9), with $n_{\tilde{x}}=n_{x}+n_{\theta }+n_{w}\cdot (i+1)$. Then the proof of theorem 2.3 follows directly from the application of the OIC of theorem 2.2. ▪

**Remark 2.4.**

Note that, in order to build $O_{I}$ with *i* Lie derivatives, the augmented state vector $\tilde{x}$ must include *w*^(*i*)^. As can be noticed from (2.8) and (2.9), its time derivative, *w*^(*i*+1)^, will not appear in $O_{I}$ (for details, see electronic supplementary material, subsection S2.1). Hence this approach enables the calculation of $\text{rank}(O_{I})$ and the assessment of the FISPO condition of theorem 2.3.

In practice, we have found that the result of this test can be inconclusive, if the $O_{I}$ matrix does not have full rank after reaching the maximum number of Lie derivatives that is computationally feasible or convenient to calculate. In such cases, we may adopt an idea similar to the one introduced in the previous subsection for the characterization of the sufficiently exciting inputs, that is, set to zero the derivatives of *w*(*t*) of order higher than a given one (*i*), introducing the following assumption:

**Assumption 2.5**

The time derivatives of the unknown input vector of the system under consideration vanish above a given order *i*, that is, *w*^(*j*)^(*t*) = 0, ∀ *j* ≥ *i*.

In principle, this assumption introduces a restriction on the type of allowed inputs. The assumption that there is a finite number of non-zero input derivatives is equivalent to assuming that the unknown inputs are polynomial functions of time, $w(t)=\sum_{k=0}^{i}a_{k}\cdot t^{k}$, in which case the analysis of their observability could be performed by assessing the identifiability of the coefficients *a*_*k*_. However, in practice the method may still provide informative suggestions about generic inputs even if assumption 2.5 is made: on the one hand, if the method determines that the OIC holds for *w*^(*i*)^(*t*) = 0, with *i* = {1, 2, 3, …}, and $\text{rank}(O_{I})$ grows uniformly as more Lie derivatives are included in $O_{I}$, this suggests that the same result holds in the limit i→∞, and thus the model can be assumed to be FISPO for any infinitely differentiable input. On the other hand, if the OIC does not hold for *w*^(*i*)^(*t*) = 0, with *i* = {1, 2, 3, …}, it can be taken as an indication that the model is not FISPO for a generic unknown input *w*(*t*).

We have created a new version of the MATLAB toolbox STRIKE-GOLDD [45] that incorporates the capability of analysing models with unmeasured inputs *w*(*t*) in this way. The new version, STRIKE-GOLDD 2.1, includes as examples the models analysed in this paper and documentation for running them. It is available at GitHub (https://github.com/afvillaverde/strike-goldd_2.1) and Zenodo (https://zenodo.org/record/2649224).

### 2.4. Simultaneous input and parameter estimation problem

In a nonlinear system of ODEs, PE is usually treated as a dynamic optimization problem. In the frequentist approach, the optimal values for the unknown parameters are computed by minimizing the difference between the model's predicted output and its corresponding experimental measurements. This type of problem is often formulated as a nonlinear programming problem (NLP) subject to possible differential and algebraic constraints, i.e.:

Find *θ* to minimize

2.12 $$L(\tilde{y}\;|\;\theta )=\prod_{k=1}^{n_{\exp}}\prod_{\;j=1}^{n_{y}}\prod_{i=1}^{n_{s}}\frac{1}{\sqrt{2\pi \sigma_{ijk}^{2}}}\;e^{\left(-{\left(y_{ijk}(x(t_{i}),\theta ,u(t_{i}))-{\tilde{y}}_{ijk}\right)}^{2}/2\sigma_{ijk}^{2}\right)}.$$

Subject to:

2.13 $$\frac{dx(t)}{dt}=f(x(t),u(t),\theta ,t),$$

2.14 $$x(t_{0})=x_{0},$$

2.15 y(t)=g(x(t),u(t),θ),

2.16 ζ(x(t),u(t),θ)≤0

2.17 $$\text{and}\;\theta^{L}\leq \theta \leq \theta^{U},\;\;$$

where *L* is the cost function to be minimized, *θ* is the vector of unknown parameters and $\tilde{y}$ are the experimental measurements corresponding to the model's predictions on the observed variables *y*(*t*). The known time-dependent inputs are represented by *u*(*t*), whereas *x* are the state variables and *σ* is the standard deviation of the measurements. Note that *θ* includes any unknown initial conditions present in the vector *x*_0_. Moreover, *f* is the set of ordinary differential equations (2.13) describing the system dynamics, and *ζ* are the algebraic inequality constraints (2.16). Finally, *θ* is subject to upper and lower bounds acting as inequality constraints (2.17).

However, the above classical formulation of PE problems is assuming that the model's inputs are always known (or accurately measured). As such, it fails to take into account any uncertainty in the input measurements or the complete lack of such measurements. In order to take into account the estimation of not only the unknown model parameters but also any unknown inputs, the above PE formulation can be generalized into an *optimal tracking problem*. This type of problem, as a special case of nonlinear optimal control problem (described in [35] as IOCP-1), defines the simultaneous estimation problem of both unknown time-dependent inputs *w*(*t*) and unknown time-invariant parameters *θ*.

In contrast with the classical PE problem where the time-dependent inputs are treated as known (measured) quantities, in an *optimal tracking problem* there can be an unknown subset of inputs that is estimated, along with the model's parameters, directly from the experimental measurements. The resulting mathematical formulation of the *optimal tracking problem* needs to account for the dependencies on the unknown inputs *w*(*t*), therefore (2.12)–(2.17) are redefined as follows:

2.18 $$\min_{\theta ,w(t)}L(\tilde{y}\;|\;\left\{\theta ,w(t)\right\}).$$

Subject to:

2.19 $$\frac{dx(t)}{dt}=f(x(t),u(t),w(t),\theta ,t),$$

2.20 $$x(t_{0})=x_{0},$$

2.21 y(t)=g(x(t),u(t),w(t),θ),

2.22 ζ(x(t),u(t),w(t),θ)≤0

2.23 $$\text{and}\;\theta^{L}\leq \theta \leq \theta^{U}.\;$$

Additionally, we consider bounds for the unknown inputs of the form:

2.24 $$w^{L}\leq w(t)\leq w^{U},$$

where *L* in (2.18) with standard deviation *σ* is the likelihood function:

2.25 $$L(\tilde{y}\;|\;\left\{\theta ,w(t)\right\})=\prod_{k=1}^{n_{\exp}}\prod_{\;j=1}^{n_{y}}\prod_{i=1}^{n_{s}}\frac{1}{\sqrt{2\pi \sigma_{ijk}^{2}}}\;e^{\left(-{\left(y_{ijk}(x(t_{i}),\theta ,u(t_{i}),w(t_{i}))-{\tilde{y}}_{ijk}\right)}^{2}/2\sigma_{ijk}^{2}\right)}.$$

In the special case where Gaussian noise can be assumed, (2.18) can be expressed in the classical weighted least-squares format, with weights *ω*:

2.26 $$\min_{\theta ,w(t)}\sum_{k=1}^{n_{\exp}}\sum_{\;j=1}^{n_{y}}\sum_{i=1}^{n_{s}}\omega_{ijk}{\left(y_{ijk}(x(t_{i}),\theta ,u(t_{i}),w(t_{i}))-{\tilde{y}}_{ijk}\right)}^{2}.$$

To avoid any confusion, we remark that in the *optimal tracking* problem stated above we do not seek the inference of the underlying optimality principles, as considered in the more general *inverse optimal control* formulation (see [35] and references therein). In other words, the problem considered here is restricted to estimating the unknown inputs and parameters of the model that best explain (fit) the available data.

Here we solved this optimal tracking problem using the control discretization method for nonlinear optimal control proposed in [35]. In the electronic supplementary material, we provide a brief overview of the numerical methods available to solve this class of problems, along with implementation details and remarks about our numerical strategy. The code to reproduce the estimation results presented in this work is available at https://zenodo.org/record/2542798 and is implemented using the methodology introduced in [35] as part of the AMIGO2 toolbox [36] for MATLAB.

## 3. Results and discussion

### 3.1. Two-compartment model (C2M)

In [50], the following two-compartment model of a physiological system was analysed

3.1 $$\begin{array}{rl} {\dot{x}}_{1}(t) & =-(k_{1e}+k_{12})\cdot x_{1}(t)+k_{21}\cdot x_{2}(t)+b\cdot u(t),\\ {\dot{x}}_{2}(t) & =k_{12}\cdot x_{1}(t)-k_{21}\cdot x_{2}(t)\\ \;\text{and}\;y(t) & =x_{1}(t), \end{array}\}$$

where each state (*x*_1_, *x*_2_) corresponds to a compartment, *θ* = (*k*_1*e*_, *k*_12_, *k*_21_, *b*) is the unknown parameter vector, and the initial condition of the unmeasured state, *x*_2_(0), is also unknown. It was shown in [50] that this model is structurally identifiable and observable for a known input such that $\dot{u}(t)\neq 0$.

Let us now consider the unknown input case. If both *b* and *u*(*t*) are unknown, obviously only their product can be estimated. Thus, we reformulate the model by introducing *w*(*t*) = *b* · *u*(*t*) as the unknown input to estimate:

3.2 $$C2M:\;\;\left\{ \begin{array}{rl} {\dot{x}}_{1}(t) & =-(k_{1e}+k_{12})\cdot x_{1}(t)+k_{21}\cdot x_{2}(t)+w(t),\\ {\dot{x}}_{2}(t) & =k_{12}\cdot x_{1}(t)-k_{21}\cdot x_{2}(t),\\ \;y(t) & =x_{1}(t). \end{array}\right.$$

The FISPO analysis of model C2M (3.2) yields that it is not observable. In fact, neither *θ* = (*k*_1*e*_, *k*_12_, *k*_21_), nor *x*_2_(*t*), nor *w*(*t*) are observable. To obtain an observable model, we need to fix one of the parameters (*k*_1*e*_, *k*_12_, *k*_21_). Thus, if we assume that the degradation constant *k*_1*e*_ is known, the FISPO analysis determines that the model is fully observable: the unmeasured state *x*_2_(*t*) is observable, the unknown parameters (*k*_12_, *k*_21_) are structurally identifiable, and the unknown input *w*(*t*) is reconstructible.

Next, we validate this result by showing that it is indeed possible to infer these values from *y*(*t*) using the optimization procedure described in §2.4. To this end, we generate pseudo-experimental datasets, both without and with the addition of noise. In the noisy case, we considered the standard deviations (*σ*) present in the likelihood function of equation (2.25) as known. In the noiseless case, we used a least-squares cost function (2.26). To improve the numerical conditioning of our problem, we use a multi-experimental (six experiments) scheme for the estimation of the noisy subcase. More details on the experimental scheme and the problem set-up can be found in the electronic supplementary material.

A comparison between the reconstruction of the system in these two simple subcases can provide useful insight. The noiseless data represent an almost ideal estimation scenario, where practical or numerical conditioning is almost perfect. As a result, the reconstruction of the noiseless subcase should approximate the theoretically possible level. Therefore, we consider the full system reconstruction from noiseless data a numerical validation of the FISPO results. On the other hand, the inclusion of noise in the data can transform and possibly even deform the solution space. This could result in the true solution no longer coinciding with the global optimal solution, making it harder or even impossible to identify the true solution without assuming any prior knowledge about it. This effect is common in realistic scenarios, although it is often overlooked. Comparing the performance of our methodology with and without noise in the data, we attempt to illustrate the possible issues of practical reconstructibility in a more realistic scenario.

**Figure 2.**
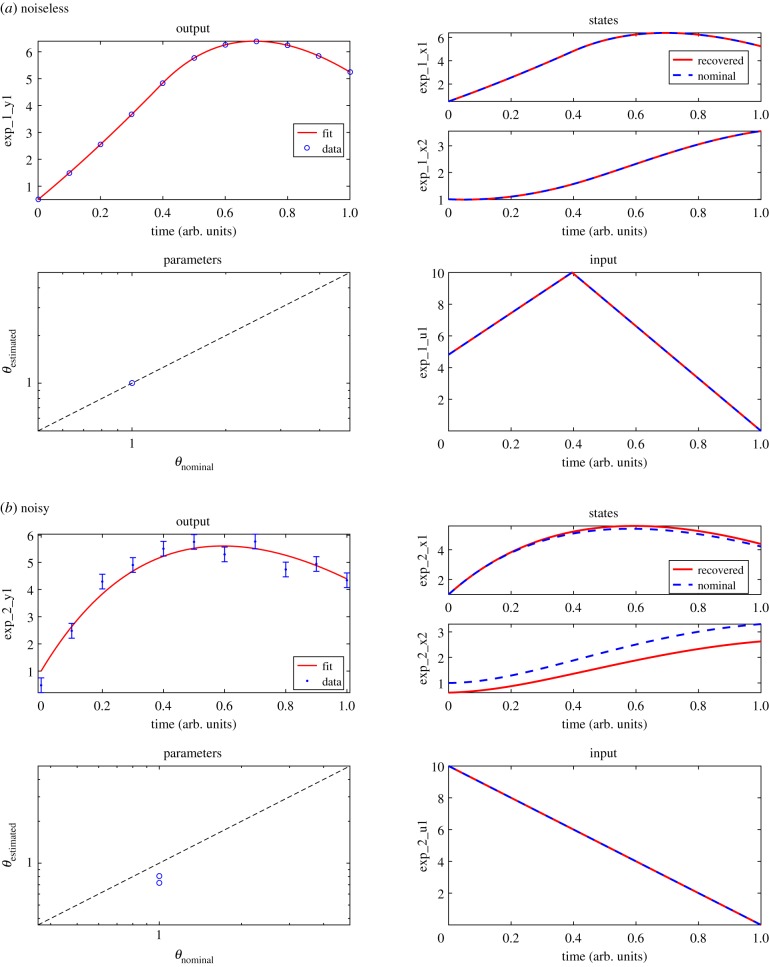
Case study C2M: results of the full system reconstruction. The top half figures (*a*) show the noiseless subcase. The bottom half figures (*b*) correspond to one of the six experiments used in the estimation of the noisy subcase; the remaining experiments are given in the electronic supplementary material. In both noiseless and noisy cases, the upper left subplots show the fit of the model output to time-series synthetic data. The remaining subplots show the estimated states, parameters and inputs. Note that the ‘Parameters’ subplot of the noiseless case shows two parameters, although their markers overlap. (Online version in colour.)

In figure 2, the results of the full system reconstruction for both subcases are presented. For the noiseless subcase (*a*), we verify that the reconstruction of all the problem unknowns is perfect, with the two unknown parameters as well as the unobserved initial condition and the ramp-like input accurately recovered. The inference becomes more challenging with the addition of noise, yet not impossible. On the one hand, the unknown input is still reliably reconstructed. On the other hand, the noisy readings of the output (*y*(*t*) = *x*_1_(*t*)) lead to a slight overestimation of the measured state, *x*_1_(*t*), which is compensated by underestimations of the unmeasured state, *x*_2_(*t*), as well as the two unknown parameters, *k*_12_ and *k*_21_. The decreased accuracy of the estimations of these quantities suggests a certain degree of correlation among them that prevents their perfect identification in the presence of noise-corrupted data. However, even in this noisy scenario the methodology achieves good system reconstruction without the use of any prior knowledge. The figures showing the reconstruction in the rest of the six experiments are given in the electronic supplementary material.

### 3.2. Genetic toggle switch

The second case study is the genetic toggle switch that was recently presented in [51] and further analysed in [52]:

3.3 $$\mathrm{TS}:\;\left\{ \begin{array}{rl} {\dot{x}}_{1}(t) & =k_{01}+\frac{k_{1}}{1+{\left(x_{2}(t)/\left(1+{\left(\mathrm{atc}(t)/\theta_{\mathrm{atc}}\right)}^{n_{\mathrm{atc}}}\right)\right)}^{n_{TetR}}}-x_{1}(t),\\ {\dot{x}}_{2}(t) & =k_{02}+\frac{k_{2}}{1+{\left(x_{1}(t)/\left(1+{\left(\mathrm{IPTG}(t)/\theta_{\mathrm{IPTG}}\right)}^{n_{\mathrm{IPTG}}}\right)\right)}^{n_{LacI}}}-x_{2}(t),\\ y_{1}(t) & =x_{1}(t),\\ y_{2}(t) & =x_{2}(t). \end{array}\right.$$

Here, the states *x*_1_(*t*) and *x*_2_(*t*) are dimensionless variables defined from the protein concentrations, and the inputs are the inducer molecules aTc(*t*) and IPTG(*t*). For details on the derivation of equations (3.3), see [52].

If both inputs are known, the OIC test reveals that the model is structurally identifiable as long as neither input is constant, i.e. the inputs must consist of a ramp to enable identifiability. Specifically, if *u*_1_ = aTc is constant, *n*_aTc_ and *θ*_aTc_ are structurally unidentifiable, and if *u*_2_ = IPTG is constant, *n*_IPTG_ and *θ*_IPTG_ are structurally unidentifiable. It has been recently noted that in some cases a time-varying input is equivalent, for observability purposes, to a piecewise constant input with several steps, or to several experiments with constant input [53]. For the application of this idea in STRIKE-GOLDD, see [50]. Since this is the case for this example, in the numerical experiments with noisy data we illustrated this possibility using four constant input experiments instead of an experiment with a ramp input.

If the inputs are unknown, a simple visual inspection of the equations reveals that they are also unobservable, and at least the aforementioned parameters are unidentifiable. In such scenario, the model is overparametrized, and it is not possible to infer aTc, *n*_aTc_, *θ*_aTc_, IPTG, *n*_IPTG_ and *θ*_IPTG_. Therefore, we redefine the (unknown) inputs so as to incorporate these parameters:

3.4 $$w_{1}(t)={\left(\frac{\mathrm{atc}(t)}{\theta_{\mathrm{atc}}}\right)}^{n_{\mathrm{atc}}}\;\text{and}\;w_{2}(t)={\left(\frac{\mathrm{IPTG}(t)}{\theta_{\mathrm{IPTG}}}\right)}^{n_{\mathrm{IPTG}}}.$$

The resulting model is

3.5 $$\mathrm{TS}:\;\left\{ \begin{array}{rl} {\dot{x}}_{1}(t) & =k_{01}+\frac{k_{1}}{1+{\left(x_{2}(t)/\left(1+w_{1}(t)\right)\right)}^{n_{TetR}}}-x_{1}(t),\\ {\dot{x}}_{2}(t) & =k_{02}+\frac{k_{2}}{1+{\left(x_{1}(t)/\left(1+w_{2}(t)\right)\right)}^{n_{LacI}}}-x_{2}(t),\\ y_{1}(t) & =x_{1}(t),\\ y_{2}(t) & =x_{2}(t), \end{array}\right.$$

where the unknown parameter vector is *θ* = [*k*_01_, *k*_1_, *n*_*TetR*_, *k*_02_, *k*_2_, *n*_*LacI*_]. The FISPO analysis of the TS model in (3.5) yields that it is fully observable, both with constant and time-varying unknown inputs.

In order to numerically test these theoretical results, we generated pseudo-experimental data and used them in order to reconstruct all six model parameters as well as the two inputs. We considered noiseless and noisy datasets as two subcases of the estimation problem. In a similar way as for the C2M model of §3.1, we used a multi-experimental scheme (four experiments) to improve the numerical conditioning of the noisy estimation problem. Details of the experimental scheme and the estimation problem's set-up can be found in the electronic supplementary material.

**Figure 3.**
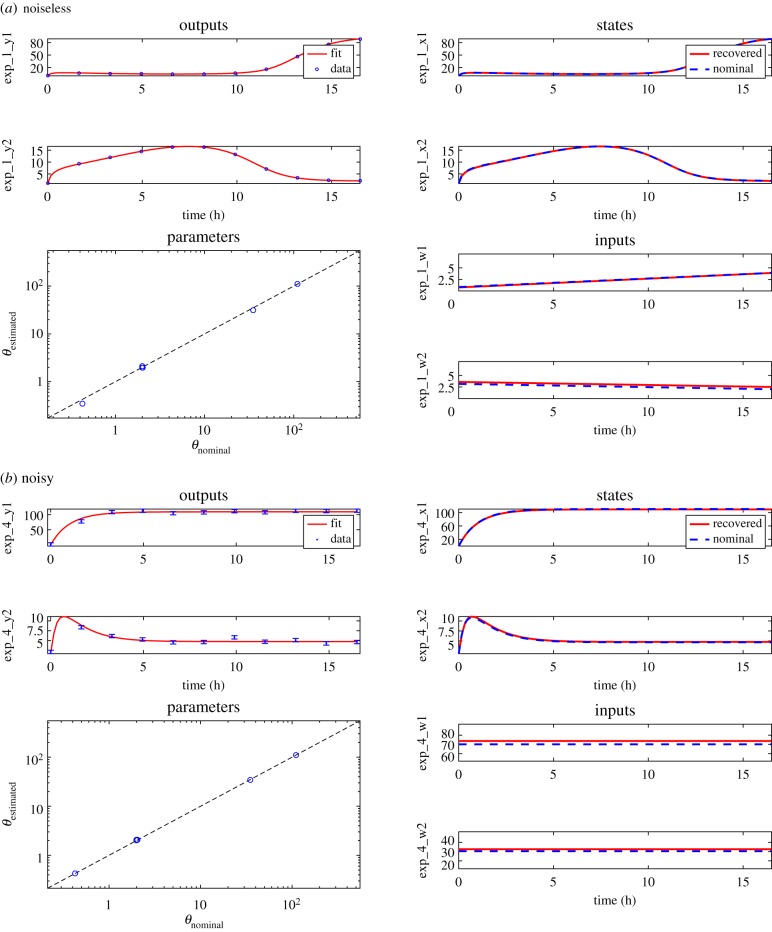
Case study TS: results of the full system reconstruction. The top half figures (*a*) show the noiseless subcase. The bottom half figures (*b*) correspond to one of the four experiments used in the estimation of the noisy subcase; the remaining experiments are given in the electronic supplementary material. In both noiseless and noisy cases, the upper left subplots show the fit of the model output to time-series synthetic data. The remaining subplots show the estimated states, parameters and inputs. (Online version in colour.)

Figure 3 shows the system reconstruction results for both the noiseless and the noisy subcases. The same remarks made in §3.1 apply here. The perfect reconstruction in the noiseless subcase (figure 3*a*) can be considered as validation of the FISPO analysis of the system, since it corresponds to a quasi-ideal scenario. The reconstruction of the system in the presence of noise is shown in figure 3*b*. Despite the addition of noise, it was possible to achieve a very accurate reconstruction of all the problem unknowns. Note that these results correspond to one of the four experiments used in the reconstruction. The figures corresponding to the rest of the multi-experimental scheme considered can be found in the electronic supplementary material.

### 3.3. HIV infection

Our third case study is the model of HIV dynamics analysed in [15,54], which is given by

3.6 $$\text{HIV}:\;\;\left\{ \begin{array}{rl} {\dot{T}}_{U}(t) & =\lambda -\rho T_{U}(t)-\eta (t)T_{U}(t)V(t),\\ {\dot{T}}_{I}(t) & =\eta (t)T_{U}(t)V(t)-\delta T_{I}(t),\\ \dot{V}(t) & =N\delta T_{I}(t)-cV(t),\\ y_{1}(t) & =V(t),\\ y_{2}(t) & =T_{I}(t)+T_{U}(t), \end{array}\right.$$

where *T*_*U*_ and *T*_*I*_ are the concentrations of uninfected and infected cells, respectively, and *V* is the viral load. The time-varying infection rate, *η*(*t*), as well as the constant parameters (λ, *ρ*, *δ*, *N*, *c*), are unknown. The FISPO analysis of this model reveals that it is fully observable even with unknown inputs, both constant and time-varying.

In order to numerically test the FISPO results, we solved the synthetic optimal control problem. We used the nominal parameter values and the *η* cosine profile taken from the literature (values provided in the electronic supplementary material) to generate pseudo-experimental data with and without noise. We then tried to reconstruct the system by approximating *η* with a number of equidistant piece-wise linear elements (ramps), assuming that no prior knowledge on the true *η* profile is available. Detailed information regarding the experimental scheme considered and the problem set-up are given in the electronic supplementary material.

Note that, as we have seen in the previous case studies, the use of a multi-experimental scheme can be very helpful in the estimation of global unknown variables (i.e. parameters that are the same in all different experiments) if there are practical identifiability issues. However, in this case study, we refrained from considering a multi-experimental scheme in the estimation. The reason is that considering multiple experiments in this case would represent the inclusion of samples from different patients in the same model calibration, which would not allow the estimation of patient specific parameters. Therefore, in this case study we used only one experiment.

**Figure 4.**
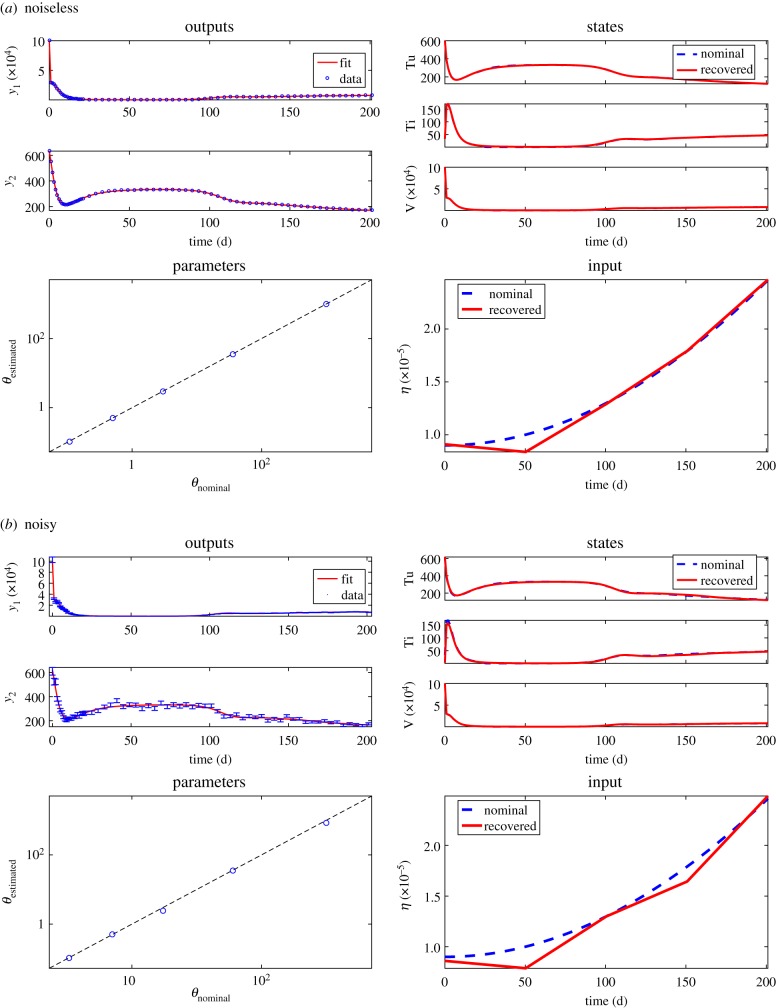
Case study HIV: results of the full system reconstruction for the noiseless (*a*) and noisy (*b*) estimations. In both noiseless and noisy cases the upper left subplots show the fit of the model output to time-series synthetic data. The remaining subplots show the estimated states, parameters and inputs. (Online version in colour.)

Despite this pseudo-experimental constraint, it was possible to obtain good results. Starting from one piece-wise linear element and iteratively re-optimizing and duplicating the number of elements, a good inference of all problem unknowns was already obtained with four piece-wise linear elements in both the noiseless and noisy subcases, as shown in figure 4. It is interesting to note that, due to the large differences in magnitude between the input and the states, the input is reconstructed with less accuracy than the states, in relative terms. In addition to validating the results of the FISPO analysis, this example illustrates the ability of the optimal tracking methodology to reconstruct inputs of arbitrary shape without any prior knowledge and with great accuracy, even in a large search space.

## 4. Conclusion

An important desirable feature of a dynamic model is the ability to infer the values of its unknown variables indirectly, by measuring its output. In mechanistic models, the unknowns typically correspond to biologically meaningful quantities, thus this inference can yield biological insight. Historically, the biological modelling community has devoted many efforts to a particular instantiation of this problem: the estimation of parameters (i.e. unknown constant values in the model) from output measurements. Other aspects of the general inverse problem are the determination of unknown inputs and unmeasured states. These three aspects have seldom been addressed jointly.

In this paper, we have described an integrated methodology that considers the general problem of determining the values of all unknown model variables, either external or internal, and constant or time-varying. We call this property Full Input-State-Parameter Observability, or FISPO for short. Our methodology begins by analysing FISPO, that is, assessing the possibility of determining the model unknowns from the model output. This first part of the methodology is of a structural nature, and its calculations are performed symbolically. Therefore, it yields theoretical results: it detects any insufficiencies of the model structure that prevent some of the model unknowns from being determined. A positive result at this stage (i.e. that it is theoretically possible to determine the value of a particular unknown) does not guarantee that the unknown will be accurately estimated from the existing data. This question is addressed in the second part of the methodology, which deals with the actual determination of the quantities that were found to be inferable in the first part. The latter part is numerical, and yields practical results for a given experimental set-up and dataset, using optimization techniques to estimate all the parameters, states and unmeasured inputs of the model.

We have demonstrated the use of this approach with three case studies from different areas of biological modelling: a two-compartment physiological system model, a genetic toggle switch and a viral infection model. We first analysed the FISPO of these models symbolically, and then used pseudo-experimental data to show that it is indeed possible to recover the values of the observable unknowns. For each case study, a different type of unmeasured input was chosen: piecewise constant, ramp and sinusoidal. In all cases, the methodology managed to estimate the unknown inputs accurately without knowing their shape, along with the parameters and states.

A known potential limitation of the symbolic analysis approach used in the first part of the methodology is that its computational complexity increases steeply with model size, hampering its applicability to large models. This issue is also present in other symbolic methods that may be explored as an alternative, such as the differential algebra approach. Numerical approaches such as profile likelihoods could in principle be applied to provide an indication of parameter identifiability, input reconstructibility and state observability. Besides computational cost, another aspect of the symbolic analysis that deserves further exploration is the handling of unmeasured inputs. The solution adopted in this paper—i.e. including the unmeasured inputs and their derivatives as additional state variables—often requires setting an upper bound to the number of non-zero derivatives of the input. While it could be argued that this is a useful feature, since it allows assessment of how the time dependence of the inputs affects the results, it requires introducing a (mild) assumption about the shape of the input. Furthermore, the algorithm may sometimes yield inconclusive results. In future work, we will explore modifications of the algorithm to improve this aspect. We expect that it might be possible to obtain more powerful results by introducing assumptions such as linear dependence on the inputs. However, this would come at the expense of a loss of generality, and our approach is currently meant to be applicable to any analytic nonlinear system. A promising step in this direction is the algorithm by Maes *et al.* [55], which has been recently proposed for mechanical systems that are affine in all inputs.

Regarding the numerical part of the methodology, it is known that the computational cost of this type of optimization problem also increases rapidly with size. However, the increase is in practice less steep than that of the symbolic step. We did not encounter significant optimization issues with the case studies considered: all computation times were of the order of minutes using a standard personal computer.

## Supplementary Material

Supplementary information: methods and results

## Data accessibility

The data and software supporting the findings of this study are available within the results section of this paper and in public repositories. The FISPO analysis was performed with the STRIKE-GOLDD 2.1 toolbox, which is available at Zenodo (https://zenodo.org/record/2649224) and GitHub (https://github.com/afvillaverde/strike-goldd_2.1). These repositories include implementations of the case studies analysed in this paper. The code to reproduce the estimation results is available at Zenodo (https://zenodo.org/record/2542798).
