# Supplementary material for: Full observability and estimation of unknown inputs, states and parameters of nonlinear biological models
Source: J R Soc Interface. 2019 Jul 3;16(156):20190043. doi: 10.1098/rsif.2019.0043 (PMC6685009; doi:10.1098/rsif.2019.0043)
Supplement: Supplementary information: methods and results [file rsif20190043supp1.pdf]

SUPPLEMENTARY FILE:
Full observability and estimation of unknown inputs, states, and parameters of nonlinear biological models

Alejandro F. Villaverde[1 *], Nikolaos Tsiantis[1, 2 *], and Julio R. Banga[1]

[1]*Bioprocess Engineering Group, IIM-CSIC, Vigo, Galicia, Spain*
[2]*Department of Chemical Engineering, University of Vigo, Vigo, Galicia, Spain*
[*]*Joint first authors*

Contents

1 Additional supplementary material **1**

2 Supplementary information: Methods **1**
2.1 On the feasibility of the FISPO analysis without Assumption 1 1
2.2 On the implications of Assumption 1 for the FISPO analysis 2
2.3 Numerical solution of nonlinear optimal control problems 3
2.3.1 Control vector parameterisation 3
2.4 Implementation 4
2.4.1 Remarks on implemented case studies 4
2.5 Remarks on input estimation 5

3 Supplementary information: Results **5**
3.1 Two-compartment model (C2M) 5
3.1.1 FISPO analysis 5
3.1.2 Numerical estimation 7
3.2 Genetic toggle switch (TS) 11
3.2.1 FISPO analysis 11
3.2.2 Numerical estimation 12
3.3 HIV infection (HIV) 14
3.3.1 FISPO analysis 14
3.3.2 Numerical estimation 15

4 References **17**

1 Additional supplementary material

In addition to this document, the following accompanying files are available:

- The FISPO analysis is performed with the MATLAB toolbox STRIKE-GOLDD 2.1, which includes implementations of the case studies analysed in the main paper. It can be downloaded from Zenodo (`https://zenodo.org/record/2649224`). The latest version of the toolbox is also available in GitHub (`https://github.com/afvillaverde/strike-goldd_2.1`).
- The numerical estimation results can be reproduced using the MATLAB input files available at Zenodo (`https://doi.org/10.5281/zenodo.2542798`).

2 Supplementary information: Methods

2.1 On the feasibility of the FISPO analysis without Assumption 1

This subsection shows that the FISPO analysis can be performed without Assumption 1, i.e. without setting to zero the $(i+1)^{\text{th}}$ time derivative of the unknown input, $w^{(i+1)}(t)$. To this end we show that $w^{(i+1)}(t)$ does not appear in $\mathcal{O}_{\mathcal{I}}$ when this matrix is built with i Lie derivatives.

Assume we build $\mathcal{O}_{\mathcal{I}}$ with i Lie derivatives. The augmented state vector and its dynamics are:

$$\tilde{x}(t) = \begin{bmatrix} x(t) \\ \theta \\ w(t) \\ \dot{w}(t) \\ \ddot{w}(t) \\ \vdots \\ w^{(i-1)}(t) \\ w^{(i)}(t) \end{bmatrix}, \qquad \tilde{f}(t) = \begin{bmatrix} f(\tilde{x}(t), u(t)) \\ 0 \\ \dot{w}(t) \\ \ddot{w}(t) \\ \dddot{w}(t) \\ \vdots \\ w^{(i)}(t) \\ w^{(i+1)}(t) \end{bmatrix}, \tag{S.1}$$

Note that the output function $g(u,w,x,\theta)$ is still a function only of $\{u,w,x,\theta\}$, not of any derivatives of u or w. Hence the first Lie derivative is:

$$L_{\tilde{f}}g(u,w,x,\theta) = \frac{\partial g(u,w,x,\theta)}{\partial \tilde{x}}\tilde{f}(\tilde{x},u) + \sum_{j=0}^{j=\infty} \frac{\partial g(u,w,x,\theta)}{\partial u^{(j)}} u^{(j+1)}. \tag{S.2}$$

Note that $g(u,w,x,\theta)$ may depend on w, but not on $\dot{w}, \ddot{w}, \ldots$. Therefore, the only elements of the vector $\frac{\partial g(u,w,x,\theta)}{\partial \tilde{x}}$ that can be non-zero are the ones corresponding to the partial derivatives with respect to $\{w,x,\theta\}$. Thus, the product $\frac{\partial g(u,w,x,\theta)}{\partial \tilde{x}}\tilde{f}(\tilde{x},u)$ may contain $\dot{w}$ but not higher order derivatives such as $\ddot{w}$, since the elements of the vector $\tilde{f}$ where those derivatives appear are multiplied by zeros.

As for the term $\sum_{j=0}^{j=\infty} \frac{\partial g(u,w,x,\theta)}{\partial u^{(j)}} u^{(j+1)}$, it may be a function of $\{u,w,x,\theta,\dot{u}\}$ (but not of higher derivatives of u or w).

Therefore, the Lie derivative of order one, $L_{\tilde{f}}g$, can be a function of $\{u, \dot{u}, w, \dot{w}, x, \theta\}$, but not of derivatives of $\{u, w\}$ of order higher than one.

This result can be easily generalized to Lie derivatives of order higher than one, which are obtained recursively as:

$$L_f^i g(\tilde{x}, u) = \frac{\partial L_f^{i-1} g(\tilde{x}, u)}{\partial \tilde{x}} f(\tilde{x}, u) + \sum_{j=0}^{j=\infty} \frac{\partial L_f^{i-1} g(\tilde{x}, u)}{\partial u^{(j)}} u^{(j+1)}. \tag{S.3}$$

Following the same reasoning as above, it can be readily seen that the Lie derivative of order *i* may contain derivatives of $\{u, w\}$ up to order *i* or lower.

As a consequence, a matrix $\mathcal{O}_{\mathcal{I}}$ built with *i* Lie derivatives does not contain $w^{(i+1)}$, even though $w^{(i+1)}$ appears in $\tilde{f}$. Hence it is not necessary to assume that $w^{(i+1)} = 0$ in order to calculate $\text{rank}(\mathcal{O}_{\mathcal{I}})$.

2.2 On the implications of Assumption 1 for the FISPO analysis

This subsection discusses the implications of the following assumption that can be made when applying the FISPO analysis method:

Assumption 1. The time derivatives of the unknown input vector of the system under consideration vanish above a given order *i*, that is, $w^{(j)}(t) = 0, \ \forall j \geq i$.

Let us first consider a typical scenario, in which the analysis of a model reveals that it is FISPO for $i = \{1, 2, 3, 4, 5, \ldots\}$. This is the case of the C2M analysed in the paper, which is computationally cheap to analyse even for large values of *i*, so we can establish that it is FISPO even for $i = 15$ (it would be feasible for larger values too, but we stopped the calculations here arbitrarily; see subsection 3.1.1 of this supplementary information for more details). In this case we know for a fact that the model is FISPO for $i = \{1, 2, 3, 4, 5, \ldots, 15\}$, that is, for all unknown inputs whose 15th and higher order derivatives are zero. It is reasonable to assume that this will be the case as *i* keeps growing (in fact, for small models such as this, this might be determined by inspecting the $\mathcal{O}_I$ matrix, but it will typically not be the case for more complex models). Therefore, we may assume that the model is FISPO for *any* unknown input. In doing so we might be overestimating observability: according to our assumptions, it would be conceivable that two different inputs whose 16th derivatives are nonzero produce the same output. However, it should be taken into account that in reality the unknown inputs to biological systems are not ideal mathematical functions. It is rather the other way around: they can be modelled, or approximated, using ideal mathematical functions, which in most cases can be approximated very accurately with low order polynomials. The result obtained for the C2M model tells us that, as long as the input can be accurately modeled with a polynomial of order equal or smaller than 15, it will be reconstructible. Theoretically, there is a chance that we would be reconstructing the 'wrong' polynomial, but the error in this estimation would come from the terms of order 15 and higher, so it is highly unlikely that it will be distinguishable from noise. Of course, for models that are computationally more expensive it may be necessary to set a much lower limit to the number of nonzero derivatives, *i*. However, even if it is only 4 or 5 it allows for a realistic representation of most biological inputs. Therefore, while there is a risk of **overestimating observability**, we believe that it should be taken more as a theoretical caution than as an actual limitation in practice.

We may also consider a different scenario: imagine that for low values of *i* the method yields that a model is not FISPO, but the rank of the $\mathcal{O}_I$ matrix keeps growing with *i*, allowing for a

theoretical chance that it will eventually have full rank with sufficient derivatives. However, due to computational limitations we are forced to stop the calculations before that happens. If we interpret these results as an indication that the model is not FISPO, we might be **underestimating the observability**. However, this would be a highly counter-intuitive situation, in which having unknown inputs of higher complexity would *improve* observability. We have never encountered it in any of the models we analysed so far. Hence we believe that the risk of underestimating observability is not a real concern. (Note that this scenario is not the same as the one in which the number of Lie derivatives is not sufficient to produce a result. In this case the method yields an inconclusive result; the STRIKE-GOLDD toolbox reports that the FISPO analysis could not be completed. Of course this should not be interpreted as a sign or observability nor unobservability.)

2.3 Numerical solution of nonlinear optimal control problems

The simultaneous input and parameter estimation problem can be formulated as an optimal control problem (see main text). Methods for the numerical solution of nonlinear optimal control problems can be classified under three main categories: dynamic programming, indirect and direct approaches. Dynamic programming [5, 6] suffers from the so-called *curse of dimensionality*, so the latter two are the most promising strategies for problems of realistic complexity. Indirect approaches are based on the transformation of the original optimal control problem into a multi-point boundary value problem using Pontryagin's necessary conditions [9, 13]. Direct methods transform the optimal control problem into a nonlinear programming problem (NLP). They are based on the discretisation of either the control, in which case they are known as "sequential" strategy [4, 17], or both the control and the states, in which case they are known as "simultaneous" strategy [7, 8].

In the sequential strategy, also known as control vector parameterisation (CVP), the controls are approximated piecewise, usually by a low order polynomial, the coefficients of which are the decision variables. The problem is transformed in an outer NLP problem optimising the decision variables provided in the inner initial value problem (IVP) where the dynamic system is integrated. In the simultaneous strategy, also known as complete parameterisation approach, both states and controls are parameterised by dividing the whole time domain into small intervals. This is done either using multiple shooting, where similarly to the sequential approach, the problem is integrated separately in each interval and linked with the rest through equality constraints, or by a collocation approach, where the solution of the dynamic system is being coupled with the optimisation problem.

The simultaneous collocation strategy leads to larger optimisation problems but has the advantage of avoiding the repeated integration of the system dynamics at each iteration. The Control vector parameterisation strategy is easier to apply to arbitrary nonlinear dynamics, and relies on well tested initial value solvers. Here, we have chosen the control vector parameterisation approach (sequential strategy).

2.3.1 Control vector parameterisation

The control vector parameterisation (CVP) approach proceeds by dividing the time horizon into a number of elements (ρ). Each of the control variables ($j = 1 \ldots n_u$) are then approximated within

each interval ($i = 1 \dots \rho$) by means of some basis functions (usually, low order Lagrange polynomials [18]) as follows:

$$u_j^{(i)}(t) = \sum_{k=1}^{M_j} u_{ijk} \ell_k^{(M_j)}(\tau^{(i)}) \quad t \in [t_{i-1}, t_i] \tag{S.4}$$

being τ the normalised time in each element i:

$$\tau^{(i)} = \frac{t - t_{i-1}}{t_i - t_{i-1}} \tag{S.5}$$

and M_j the order of the Lagrange polynomial (ℓ). In this work we will consider $M_j = 1$ or $M_j = 2$, i.e. step-wise or linear-wise control approximations.

In the CVP approach, the controls are expressed as functions of a new set of time invariant parameters corresponding to the polynomial coefficients (w). Therefore the original infinite dimensional problem is transformed into a set of non-linear programming problems, with dynamic (the model) and algebraic constraints, in which the decision variables correspond to the original unknown parameters in θ and w, which will be part of the overall set of parameters to determine.

2.4 Implementation

In order to perform the FISPO analysis, a new release of the STRIKE-GOLDD [19] toolbox for MATLAB was created for this paper. It incorporates the capability of analysing models with unmeasured inputs, $w(t)$, and includes as examples all the case studies presented in this article. This new release, STRIKE-GOLDD 2.1.2, is available at Zenodo (`https://zenodo.org/record/2649224`) and GitHub (`https://github.com/afvillaverde/strike-goldd_2.1`).

Regarding the estimation problem, the methodology was introduced in [16] and implemented as a software module (IOC add-on) for the AMIGO2 toolbox ([2]). The IOC add-on is publicly available at `https://sites.google.com/site/amigo2toolbox/home/amigo2_ioc`. The implementation of the case studies reported in this paper is available at Zenodo (`https://doi.org/10.5281/zenodo.2542798`). In all the computations we have used the eSS optimiser, a hybrid global-local optimisation meta-heuristic available in the AMIGO2 toolbox.

2.4.1 Remarks on implemented case studies

As mentioned in the previous sections, the methodology used for the numerical estimation problem of the full system reconstruction was introduced in our previous work [16]. For any further discussion on the use of this methodology or on its scalability we refer the interested reader to the aforementioned paper.

In order to enable the computational reproducibility of the estimation results presented in this article, we provide the necessary scripts that include all the settings that were used, along with the exact synthetic pseudo-experimental data sets that were generated and the CPU seed from which the computation was initialised. The latter is necessary for the reproduction of the exact numerical results, since eSS is a partially stochastic optimisation solver that is generating pseudo-random reference sets of points in the search space.

It should be noted that the majority of the numerical subcases presented in this paper were solved, as can be seen in the provided scripts, using the eSS method with the local solver FSQP [12]. This local solver is not publicly available with the AMIGO2 toolbox, since it requires a licence. Substituting FSQP with any other solver available in the AMIGO2 toolbox can result in differences in the convergence of the solver (e.g. higher CPU time requirements) and therefore affect the accurate reproduction of the presented results.

2.5 Remarks on input estimation

In the present work we have used a standard control vector parameterization (CVP) scheme [17, 18], where inputs (controls) are approximated using low order piecewise polynomials, typically piecewise constant (PWC) or piecewise linear (PWL) approximations. We refined the controls according to the following procedure:

1. We chose basis functions (PWC, PWL) and an initial coarse discretization based on prior knowledge about the smoothness of the input to be reconstructed. PWC should be chosen in the absence of prior knowledge, or when a non-smooth input is expected.

2. Since we did not assume any prior knowledge, we chose PWC and proceeded to refine the control mesh. If the final result indicated a relatively smooth profile, we considered a PWL discretization with a coarser mesh.

3. We proceeded using an iterative mesh refinement technique (successive re-optimisations increasing the control discretization, as described in [1]) until we obtained a satisfactory fit to data.

In the three case studies considered here, relatively coarse discretisations were sufficient to obtain a good fit to the data. Additional examples of the use of the above scheme for case studies with inputs of different non-smoothness can be found in [3, 11].

The above procedure works well for most problems, but if the computational cost of the mesh refinement scheme becomes very significant (e.g. cases with very wild input profiles and which are computationally very costly to evaluate, such as large-scale dynamic models), one can resort to CVP variants that introduce efficient control discretisations which are able to approximate difficult input profiles with coarser discretisations (a review of recent methods is given in [15]).

3 Supplementary information: Results

3.1 Two-compartment model (C2M)

3.1.1 FISPO analysis

We performed the FISPO analysis of three different versions of the C2M model defined in Section 3.1 of the main paper:

1. The model with known input u, as originally presented in [20] and defined by equation (25) of the main text.

2. The model with unknown input $w(t) = b \cdot u(t)$, as in eq. (26) of the main text.

3. The model with unknown input $w(t) = b \cdot u(t)$, and with the parameter k_{1e} considered as a known constant.

For each version we considered a number of different bounds on the number of nonzero derivatives that the inputs are assumed to have (i_U for the known inputs, i_W for the unknown ones), to assess their influence on the outcome of the FISPO analysis. Table S.T1 shows the results for the different options, including the CPU time of the calculations. The number of Lie derivatives required to reach a solution, as well as the corresponding rank of the generalised observability matrix O_I, are also shown.

For this case study the number of nonzero derivatives affects the result if the input is known, but not if it is unknown. For the unknown input case the results are constant for all the numbers chosen. We take this as an indication that the same result holds as $i_W \to \infty$.

Table S.T1: Results of the FISPO analyses for the C2M case study. i_U: number of nonzero derivatives of the known input (N/A: not applicable, if the model does not have known inputs); i_W: number of nonzero derivatives of the unknown input (N/A: not applicable, if the model does not have unknown inputs); #Lie: number of Lie derivatives calculated to obtain the result; CPU time (s): computation time rounded to the nearest second.

Version	i_U	i_W	#Lie	rank(O_I)	FISPO	CPU time (s)
1	0	N/A	6	5	no	1
	1	N/A	5	6	yes	1
2	N/A	0	6	5	no	2
	N/A	1	6	6	no	2
	N/A	2	7	7	no	3
	N/A	3	8	8	no	3
	N/A	4	9	9	no	5
	N/A	5	10	10	no	8
	N/A	6	11	11	no	14
	N/A	7	12	12	no	29
	N/A	8	13	13	no	59
	N/A	9	14	14	no	129
	N/A	10	15	15	no	315
3	N/A	0	4	5	yes	2
	N/A	1	5	6	yes	3
	N/A	2	6	7	yes	5
	N/A	3	7	8	yes	7
	N/A	4	8	9	yes	9
	N/A	5	9	10	yes	10
	N/A	6	10	11	yes	16
	N/A	7	11	12	yes	19
	N/A	8	12	13	yes	28
	N/A	9	13	14	yes	35
	N/A	10	14	15	yes	51
	N/A	11	15	16	yes	66
	N/A	12	16	17	yes	140
	N/A	13	17	18	yes	295
	N/A	14	18	19	yes	602
	N/A	15	19	20	yes	1225

3.1.2 Numerical estimation

For this fully observable model, the optimal tracking approach was used to reconstruct the full system. Two subcases were considered in this problem:

1. A noiseless subcase using synthetic data without any addition of noise. For this case the estimation should achieve the theoretically possible results, numerically proving or disproving

the FISPO analysis.

2. A noisy subcase, where the addition of noise to the synthetic data allows us to assess the practical reconstructibility of the model in a more realistic scenario.

The estimation problem arising from this model includes: two unknown model parameters, one unobserved initial condition (as well as the corresponding state), and one unmeasured input. The nominal parameter values were all equal to 1 with $b = 2$. The time horizon was considered in arbitrary units between 0 and 1, taking 11 samples. For the noiseless subcase no noise was added to the data, while in the noisy subcase 5% homoscedastic noise was added. In both subcases the search space considered was [0, 10] in the case of the input and a two orders of magnitude space around their respective nominal values in the case of the parameters.

The results of the numerical estimation for the noiseless subcase are given in the main text. The noiseless results approximated the theoretically possible (perfect) inference. For the noisy subcase the problem requires several experiments in order to be practically reconstructible. We used 6 different experiments with pseudo-experimental noisy data, with different input values and a different initial condition of the observed state (x_1) in each of them. In the main article we show the reconstruction results for one of the 6 experiments, and the remaining ones are shown here in Figures S1-S5. It can be noticed that after the addition of noise to the data the estimation is still good but obviously worse, highlighting the practical numerical issues that are encountered in a realistic instance of an optimal tracking.

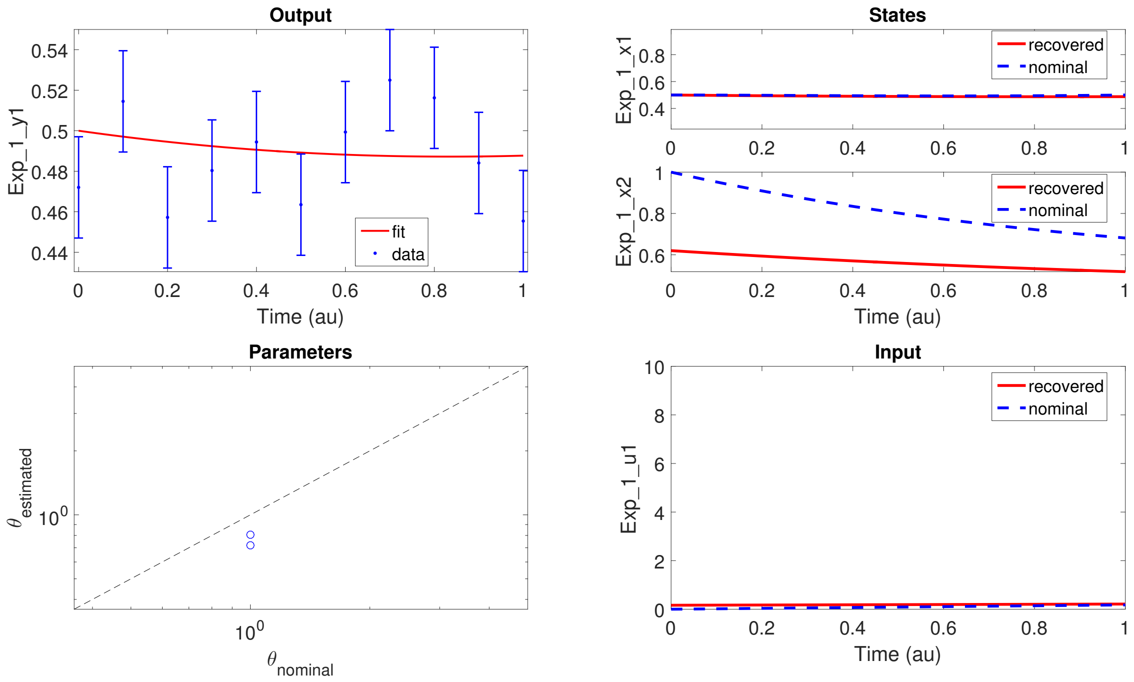

Figure S1: Case study C2M, Experiment 1: full system reconstruction with noisy time-series synthetic data, corresponding to one (Exp_1) of the 6 experimental schemes used in the estimation.

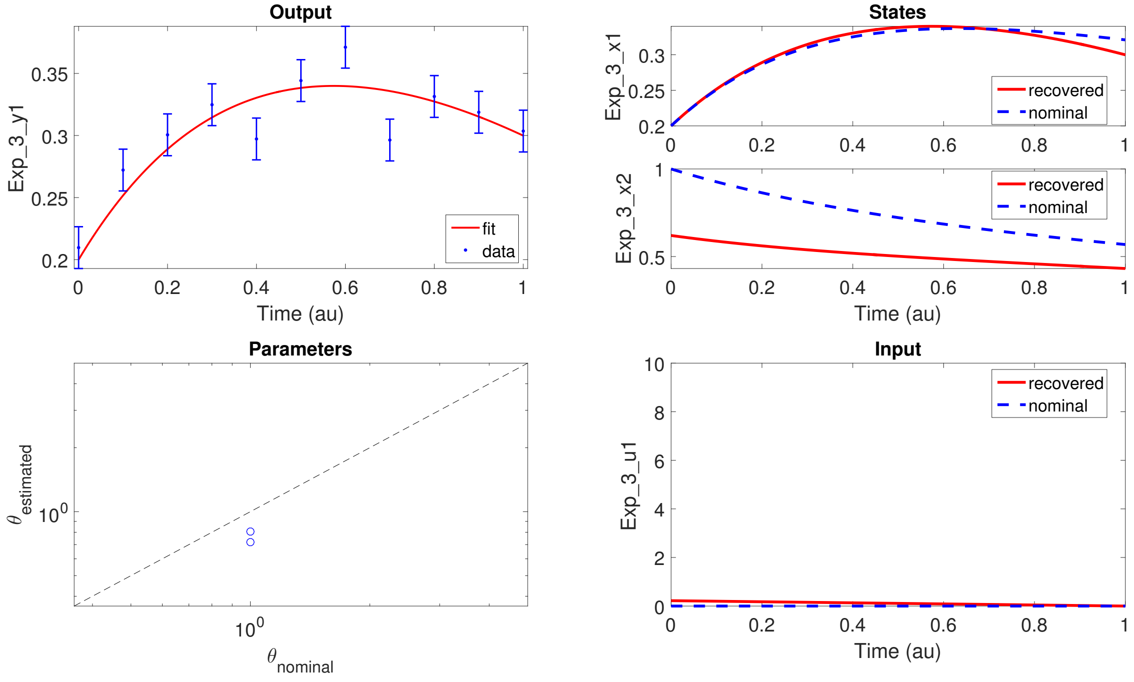

Figure S2: Case study C2M, Experiment 3: full system reconstruction with noisy time-series synthetic data, corresponding to one (Exp_3) of the 6 experimental schemes used in the estimation.

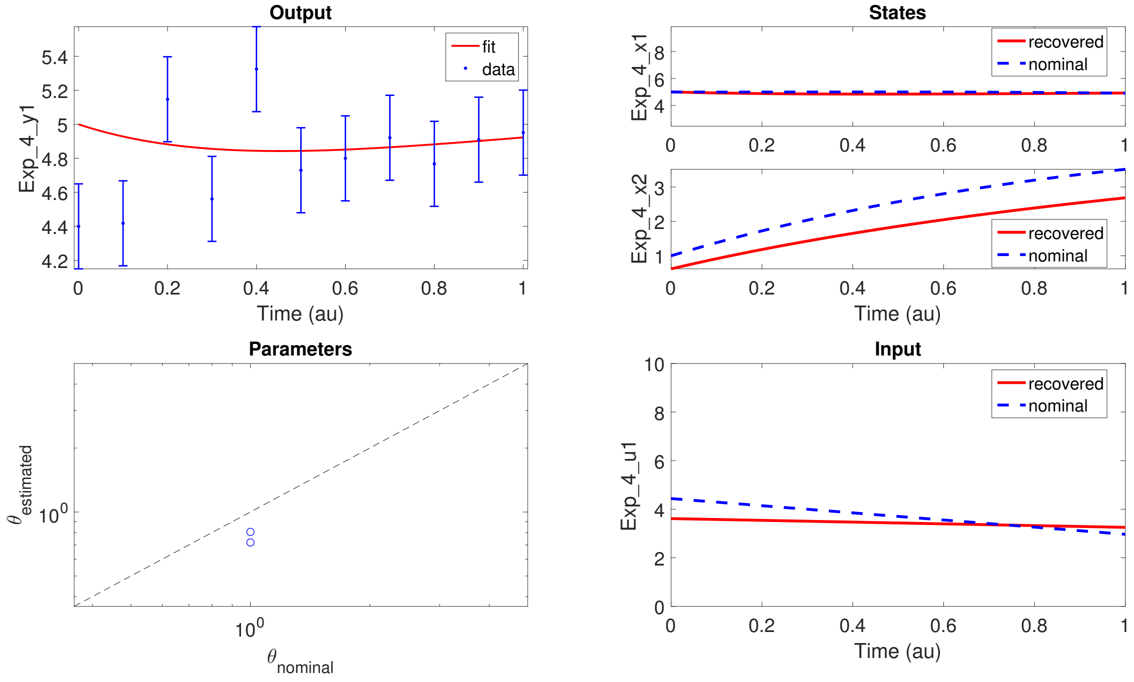

Figure S3: Case study C2M, Experiment 4: full system reconstruction with noisy time-series synthetic data, corresponding to one (Exp_4) of the 6 experimental schemes used in the estimation.

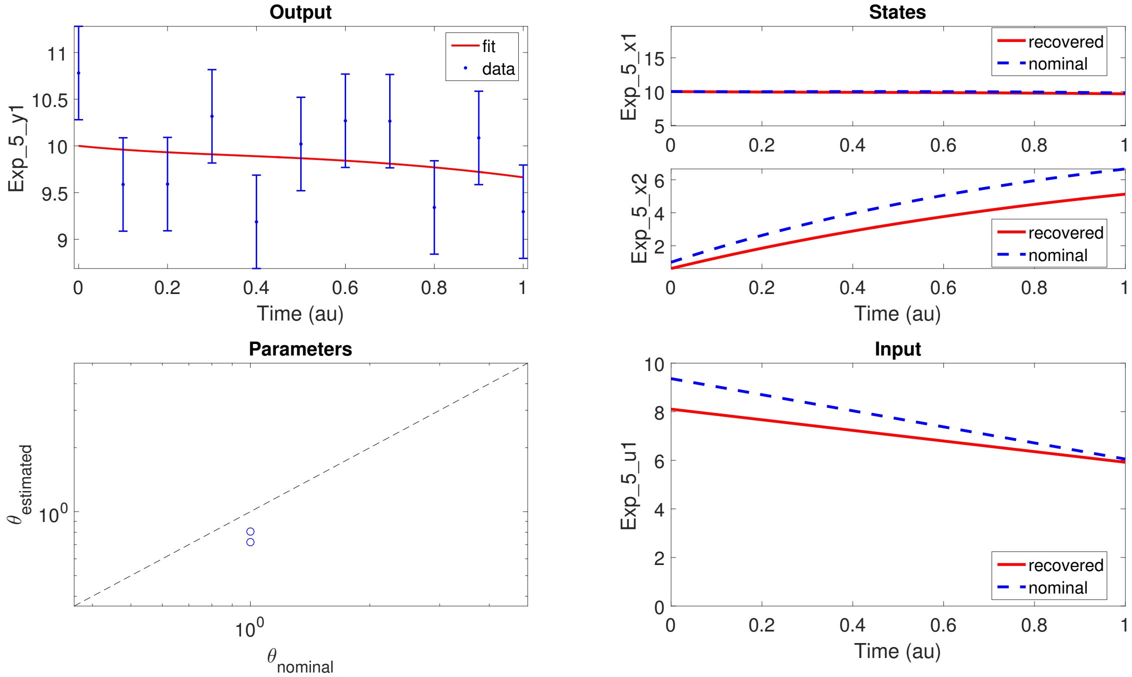

Figure S4: Case study C2M, Experiment 5: full system reconstruction with noisy time-series synthetic data, corresponding to one (Exp_5) of the 6 experimental schemes used in the estimation.

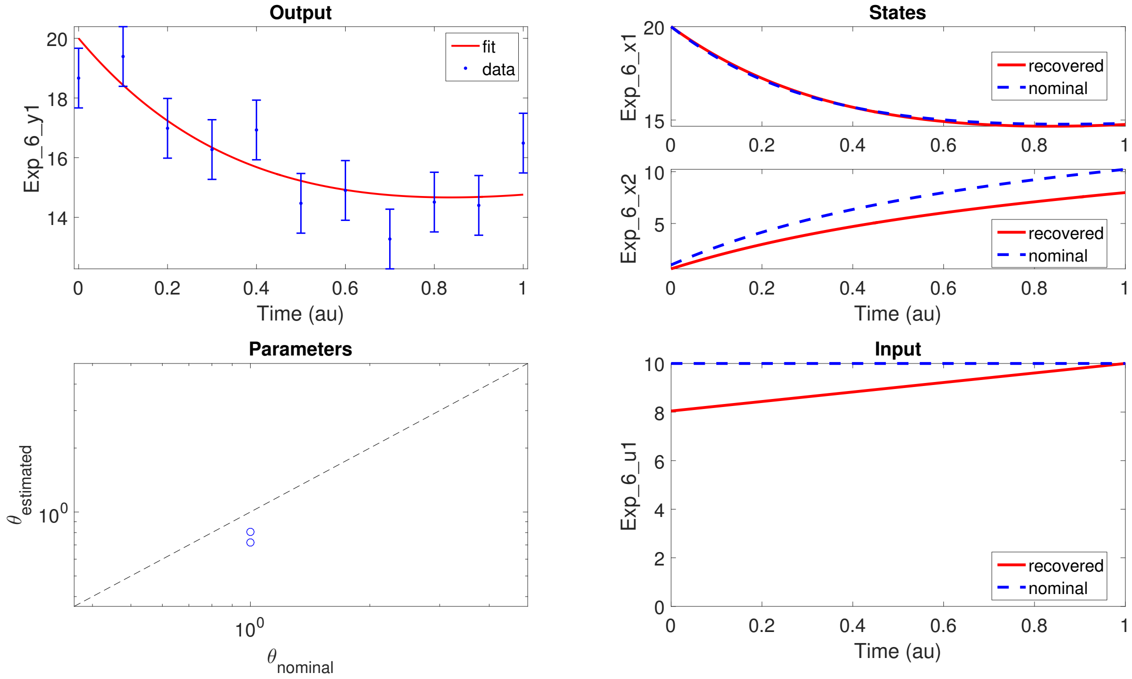

Figure S5: Case study C2M, Experiment 6: full system reconstruction with noisy time-series synthetic data, corresponding to one (Exp_6) of the 6 experimental schemes used in the estimation.

3.2 Genetic toggle switch (TS)

3.2.1 FISPO analysis

Similarly as for the C2M case study analysed in the previous subsection, we performed the FISPO analysis of two different versions of the TS model defined in Section 3.2 of the main paper:

1. The model with known inputs as in [10, 14], defined by equation (27) of the main text.
2. The reformulated model with unknown inputs defined by eqs. (28, 29) of the main text.

Again, for each model version we considered a number of different bounds on the number of nonzero derivatives that the inputs are assumed to have (i_U for the known inputs, i_W for the unknown ones), to assess their influence on the outcome of the FISPO analysis. Table S.T2 shows the results for the different options, including the CPU time of the calculations. The number of Lie derivatives required to reach a solution, as well as the corresponding rank of the generalised observability matrix O_I, are also shown.

For this model the number of nonzero derivatives affects the result for the case of known inputs, but not for the unknown inputs case. (Note that in the latter case the model formulation is different, having been reparameterised.) The model with unknown inputs keeps being FISPO as the number of nonzero input derivatives grows. We take this as an indication that the same result holds as $\{i_{W_1}, i_{W_2}\} \to \infty$.

Table S.T2: Results of the FISPO analyses for the TS case study. i_{U_1}, i_{U_2}: number of nonzero derivatives of the known inputs, u_1 and u_2 (N/A: not applicable, if the model does not have known inputs); i_{W_1}, i_{W_2}: number of nonzero derivatives of the unknown inputs, w_1 and w_2 (N/A: not applicable, if the model does not have unknown inputs); #Lie: number of Lie derivatives calculated to obtain the result; CPU time (s): computation time rounded to the nearest second.

Version	i_{U_1}	i_{U_2}	i_{W_1}	i_{W_2}	#Lie	rank(O_I)	FISPO	CPU time (s)
1	0	0	N/A	N/A	6	10	no ($n_{atc}, n_{IPTG}, \theta_{atc}, \theta_{IPTG}$ are SUI)	234
	0	1	N/A	N/A	6	11	no (n_{atc}, θ_{atc} are SUI)	1336
	1	0	N/A	N/A	6	11	no (n_{IPTG}, θ_{IPTG} are SUI)	1322
	1	1	N/A	N/A	5	12	yes	159
	1	2	N/A	N/A	5	12	yes	177
	2	1	N/A	N/A	5	12	yes	169
	2	2	N/A	N/A	5	12	yes	184
	3	3	N/A	N/A	5	12	yes	211
2	N/A	N/A	0	0	4	10	yes	16
	N/A	N/A	0	1	5	11	yes	21
	N/A	N/A	1	0	5	11	yes	21
	N/A	N/A	1	1	5	12	yes	43
	N/A	N/A	1	2	6	13	yes	694
	N/A	N/A	2	1	6	13	yes	665
	N/A	N/A	2	2	6	14	yes	762
	N/A	N/A	3	3	7	16	yes	4919

3.2.2 Numerical estimation

Following the same procedure as with the C2M case study, we perform the numerical estimation for the fully observable version of the model with unknown inputs in two synthetic subcases: (i) without and (ii) with the addition of noise to the generated data. We consider the noiseless subcase as a numerical validation of the FISPO analysis results, and the noisy subcase as a numerical validation of the IOC methodology.

The problem consists of 6 unknown model parameters and 2 unknown inputs. The nominal values of the unknown parameters are given in Table S.T3. Regarding the experimental scheme, 11 equidistant samples were assumed to be taken within a time horizon [0,16.5] in arbitrary units. The search space used in the estimation problem was a two orders of magnitude space around their respective nominal values, in the case of the time-invariant unknowns (i.e. the parameters), and of space of [0, 73.68] and [0, 121.83] in the case of the unknown inputs, w_1 and w_2, respectively. The input search space was adopted according to the reformulation to the upper bounds considered for $atc(t)$ and $IPTG(t)$ in [14] and [10].

Table S.T3: Nominal parameter values considered in TS

p_i	nominal value
k_{01}	0.42609
k_1	110.51730
n_{TetR}	2
k_{02}	2.02938
k_2	35.13053
n_{LacI}	2

In the noisy subcase, 5% homoscedastic noise was included in the pseudo-experimental data. To avoid the numerical ill-conditioning of the estimation problem we considered – as we did for the previous case study (C2M) – a multi-experimental scheme with 4 experiments that had the same experimental conditions but different nominal input values.

The main text of the article shows the results of the the full system reconstruction for the noiseless subcase for one of the four experiments used in the noisy subcase (Exp_4). The rest of the results are presented here in Figures S6-S8. They show that the full system reconstruction in the noisy case was very good, successfully inferring both the unknown model parameters and the unknown inputs in every experiment.

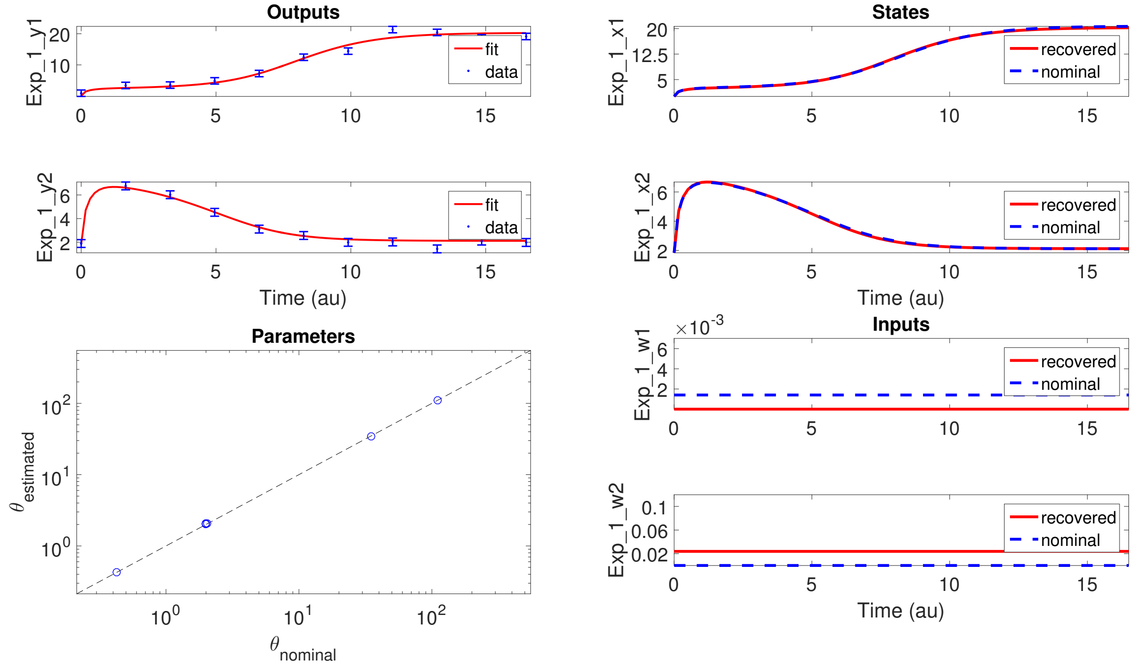

Figure S6: Case study TS, Experiment 1: full system reconstruction with noisy time-series synthetic data, corresponding to one (Exp_1) of the 4 experimental schemes used in the estimation.

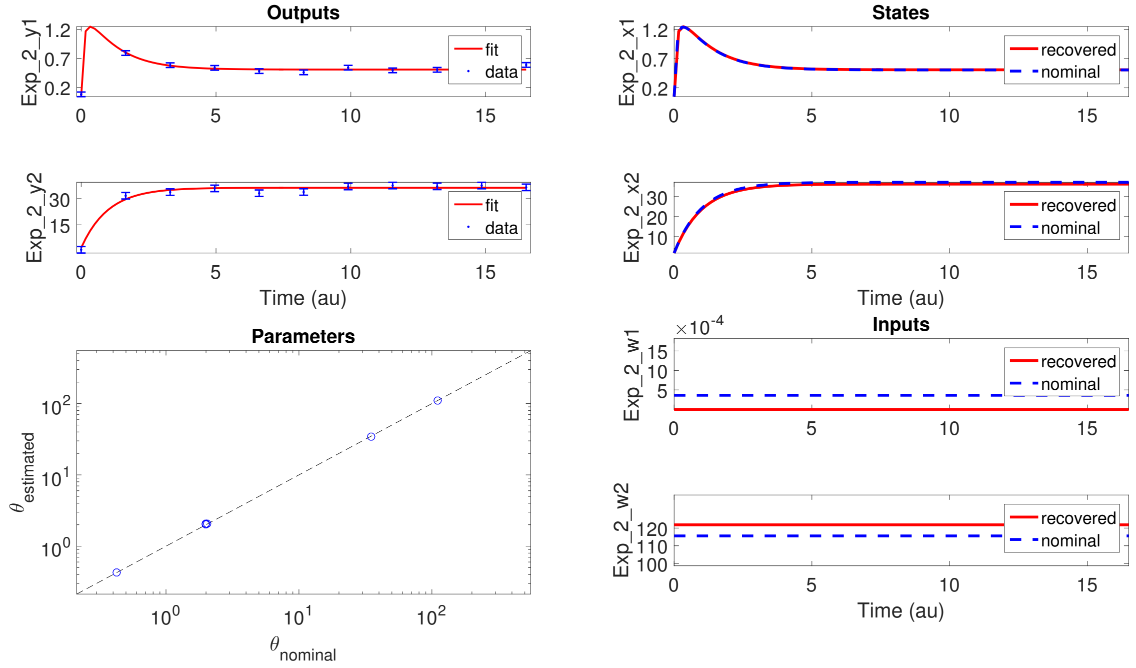

Figure S7: Case study TS, Experiment 2: full system reconstruction with noisy time-series synthetic data, corresponding to one (Exp_2) of the 4 experimental schemes used in the estimation.

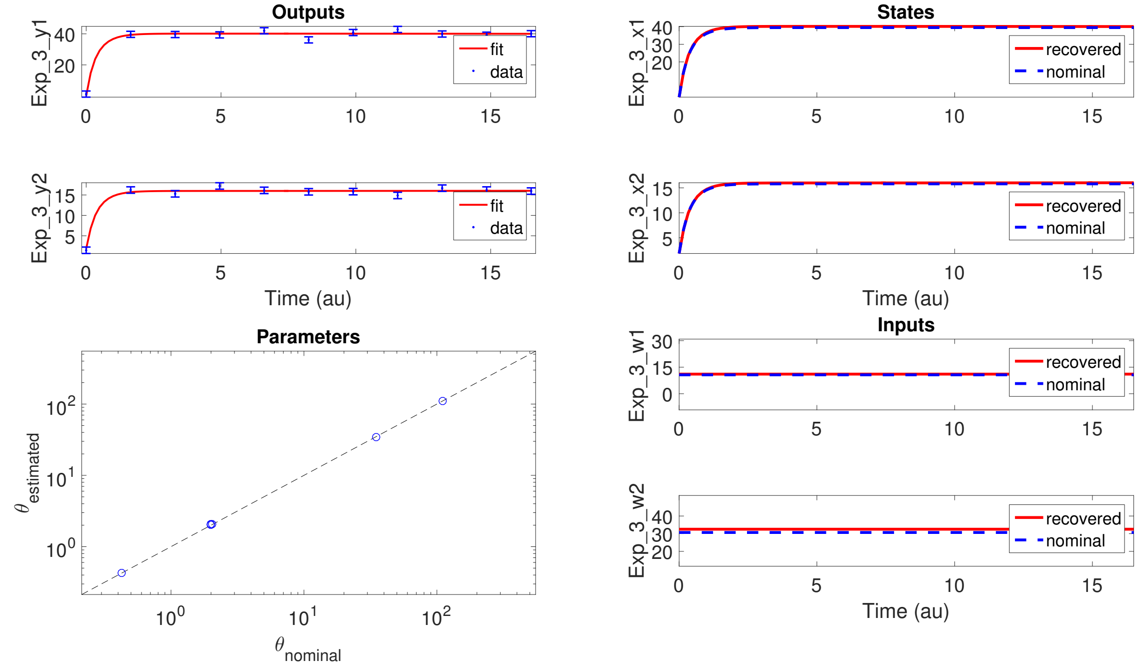

Figure S8: Case study TS, Experiment 3: full system reconstruction with noisy time-series synthetic data, corresponding to one (Exp_3) of the 4 experimental schemes used in the estimation.

3.3 HIV infection (HIV)

3.3.1 FISPO analysis

We performed the FISPO analysis of two different versions of the HIV model defined in Section 3.3, eq. (30) of the main paper:

1. The model with the time-varying infection rate, $\eta(t)$, considered as a known input.

2. The model with $\eta(t)$ considered as an unknown input.

For each model version we considered a number of different bounds on the number of nonzero derivatives that the inputs are assumed to have (i_U for the known inputs, i_W for the unknown ones), to assess their influence on the outcome of the FISPO analysis. Table S.T4 shows the results for the different options, including the CPU time of the calculations. The number of Lie derivatives required to reach a solution, as well as the corresponding rank of the generalised observability matrix O_I, are also shown.

STRIKE-GOLDD determined that the model is FISPO both with known and unknown inputs. Therefore, unlike in the other case studies, no re-formulation was required for estimating the full system. The number of nonzero derivatives did not affect the results.

To demonstrate the possibility of performing the FISPO analysis without the Assumption 1, i.e. without limiting the number of non-zero derivatives of the unknown input, we also analysed a third version of the model:

3. The model with $\eta(t)$ considered as an unknown input, and either δ or N considered as a known parameter.

As shown in table S.T4, STRIKE-GOLDD determined that this third version the model is FISPO even with an infinite number of nonzero derivatives. In contrast, for the model version 2, i.e. with all parameters unknown, the result was inconclusive: STRIKE-GOLDD reported that $\{\lambda, \rho, c\}$ are identifiable, but could not decide about the remaining variables within the maximum computation time that we allowed (5000 seconds).

Table S.T4: Results of the FISPO analyses for the HIV case study. i_U: number of nonzero derivatives of the known input (N/A: not applicable, if the model does not have known inputs); i_W: number of nonzero derivatives of the unknown input (N/A: not applicable, if the model does not have unknown inputs); #Lie: number of Lie derivatives calculated to obtain the result; CPU time (s): computation time rounded to the nearest second.

Version	i_U	i_W	#Lie	rank(O_I)	FISPO	CPU time (s)
1	0	N/A	3	8	yes	< 1
	1	N/A	3	8	yes	< 1
	2	N/A	3	8	yes	< 1
	3	N/A	3	8	yes	< 1
	4	N/A	3	8	yes	< 1
	5	N/A	3	8	yes	< 1
2	N/A	0	4	9	yes	< 1
	N/A	1	4	10	yes	< 1
	N/A	2	5	11	yes	< 1
	N/A	3	6	12	yes	2
	N/A	4	7	13	yes	7
	N/A	5	8	14	yes	41
3	N/A	∞	4	10	yes	2

3.3.2 Numerical estimation

Similarly to the previous case studies we consider (i) a noiseless and (ii) a noisy synthetic subcase in the estimation problem. However, as explained in the main article, in contrast with the previous case studies a single-experimental scheme was used in the estimation of the noisy subcase, in order to consider a realistic scenario of a patient-specific full system reconstruction of this model.

The problem includes the 5 unknown model parameters, the 2 unobserved initial conditions, and the unknown input. All nominal values considered for the problem's unknowns were taken from the literature and are presented in Table S.T5. The nominal input is found in the literature to be a cosine function of time, i.e.:

$$\eta = k(1 - 0.9\cos\frac{\pi t}{1000}) \tag{S.6}$$

Table S.T5: Nominal parameter values considered in HIV

p_i	nominal value
ρ	0.108
δ	0.5
c	3
N	1000
λ	36
k	0.00009

In the estimation problem, the time horizon considered is [0, 201], measured in days. Following a realistically possible experimental scheme we assume daily measurements for the first 20 days, and once per 4 days after that. The search space considered was $[10^{-8}, 10^{-1}]$ in the case of η, and a two orders of magnitude space around their respective nominal values in the case of the 5 unknown parameters and the 2 unobserved initial conditions. To improve the numerical conditioning of the NLP problem, scaling was used in the y_1 and η. This can be easily identified in the scripts provided.

To reconstruct the input η we approximated it with a number of equidistant piece-wise linear elements (PWL). Assuming no prior knowledge on the nominal input's shape, we utilised a re-optimisation based technique that was also used in our previous work [16] for handling arbitrary inputs. Solving the problem considering an initial crude discretisation of the input, we used this result as the initial guess for a re-optimisation of the same problem with the double of PWL elements. Iterating this solution strategy, we started from one PWL element (a ramp starting between t_0 and t_f) and increased the number of PWL to 2 and subsequently to 4. We observed that the solution using 4 PWL was already a very good reconstruction of the system. The results regarding both the noiseless and the noisy subcases are shown in the main article. Please note that, in order to reproduce those results using the scripts that are provided as supplementary files, it is necessary to apply the re-optimisation based approach described above.

4 References

[1] E. Balsa-Canto, J. R. Banga, A. A. Alonso, and V. S. Vassiliadis. Dynamic optimization of chemical and biochemical processes using restricted second-order information. *Computers & Chemical Engineering*, 25(4-6):539–546, 2001.

[2] E. Balsa-Canto, D. Henriques, A. Gábor, and J. Banga. Amigo2, a toolbox for dynamic modeling, optimization and control in systems biology. *Bioinformatics*, 32(21):3357–3359, 2016.

[3] J. R. Banga, E. Balsa-Canto, C. G. Moles, and A. A. Alonso. Dynamic optimization of bioprocesses: Efficient and robust numerical strategies. *J. Biotechnol.*, 117(4):407–419, 2005.

[4] P. I. Barton, R. J. Allgor, W. F. Feehery, and S. Galán. Dynamic optimization in a discontinuous world. *Industrial & Engineering Chemistry Research*, 37(3):966–981, 1998.

[5] R. Bellman. Dynamic programming and lagrange multipliers. *Proc. Natl. Acad. Sci. USA*, 42(10):767–769, 1956.

[6] D. P. Bertsekas. *Dynamic programming and optimal control*. Athena Scientific Belmont, MA, 1995.

[7] L. T. Biegler. Advanced optimization strategies for integrated dynamic process operations. *Computers & Chemical Engineering*, 2017. In press.

[8] L. T. Biegler, A. M. Cervantes, and A. Wächter. Advances in simultaneous strategies for dynamic process optimization. *Chem. Eng. Sci.*, 57(4):575–593, 2002.

[9] A. E. Bryson. *Applied optimal control: optimization, estimation and control*. CRC Press, 1975.

[10] D. Fiore, A. Guarino, and M. di Bernardo. Analysis and control of genetic toggle switches subject to periodic multi-input stimulation. *IEEE Control Syst. Lett.*, 3(2):278–283, 2019.

[11] T. Hirmajer, E. Balsa-Canto, and J. R. Banga. Dotcvpsb, a software toolbox for dynamic optimization in systems biology. *BMC bioinformatics*, 10(1):199, 2009.

[12] C. T. Lawrence and A. L. Tits. Nonlinear equality constraints in feasible sequential quadratic programming. *Optimization Methods and Software*, 6(4):265–282, 1996.

[13] D. Liberzon. *Calculus of variations and optimal control theory: a concise introduction*. Princeton University Press, 2012.

[14] J.-B. Lugagne, S. S. Carrillo, M. Kirch, A. Köhler, G. Batt, and P. Hersen. Balancing a genetic toggle switch by real-time feedback control and periodic forcing. *Nat. Commun.*, 8(1):1671, 2017.

[15] A. Sahlodin and P. Barton. Efficient control discretization based on turnpike theory for dynamic optimization. *Processes*, 5(4):85, 2017.

[16] N. Tsiantis, E. Balsa-Canto, and J. R. Banga. Optimality and identification of dynamic models in systems biology: an inverse optimal control framework. *Bioinformatics*, 34(14):2433–2440, 2018.

[17] V. Vassiliadis, R. Sargent, and C. Pantelides. Solution of a class of multistage dynamic optimization problems. 2. Problems with path constraints. *Ind. Eng. Chem. Res.*, 33(9):2123–2133, 1994.

[18] V. S. Vassiliadis, C. C. Pantelides, and R. W. H. Sargent. Solution of a class of multistage dynamic optimization problems. 1. Problems without path constraints. *Ind. Eng. Chem. Res.*, 33(9):2111–2122, 1994.

[19] A. F. Villaverde, A. Barreiro, and A. Papachristodoulou. Structural identifiability of dynamic systems biology models. *PLoS Comput. Biol.*, 12(10):e1005153, 2016.

[20] A. F. Villaverde, N. D. Evans, M. J. Chappell, and J. R. Banga. Input-dependent structural identifiability of nonlinear systems. *IEEE Control Syst. Lett.*, 3(2):272–277, 2019.
